# Equine Herpesvirus Type 1 Modulates Cytokine and Chemokine Profiles of Mononuclear Cells for Efficient Dissemination to Target Organs

**DOI:** 10.3390/v12090999

**Published:** 2020-09-08

**Authors:** Selvaraj Pavulraj, Mohamed Kamel, Heike Stephanowitz, Fan Liu, Johanna Plendl, Nikolaus Osterrieder, Walid Azab

**Affiliations:** 1 Institut für Virologie, Robert von Ostertag-Haus, Zentrum für Infektionsmedizin, Freie Universität Berlin, Robert-von-Ostertag-Straße 7-13, 14163 Berlin, Germany; pavulraj1vet@zedat.fu-berlin.de (S.P.); m_salah@cu.edu.eg (M.K.); no.34@fu-berlin.de (N.O.); 2 Department of Medicine and Infectious Diseases, Faculty of Veterinary Medicine, Cairo University, 12211 Cairo, Egypt; 3 Leibniz Institute of Molecular Pharmacology (FMP Berlin), Robert-Rössle-Str. 10, 13125 Berlin, Germany; Stephanowitz@fmp-berlin.de (H.S.); FLiu@fmp-berlin.de (F.L.); 4 Institut für Veterinäranatomie, Freie Universität Berlin, Koserstraße 20, 14195 Berlin, Germany; plendl.johanna@vetmed.fu-berlin.de

**Keywords:** EHV-1, pathogenesis, flow chamber assay, endothelium, equine, PBMC

## Abstract

Equine herpesvirus type 1 (EHV-1) causes encephalomyelopathy and abortion, for which cell-associated viremia and subsequent virus transfer to and replication in endothelial cells (EC) are responsible and prerequisites. Viral and cellular molecules responsible for efficient cell-to-cell spread of EHV-1 between peripheral blood mononuclear cells (PBMC) and EC remain unclear. We have generated EHV-1 mutants lacking *ORF1*, *ORF2*, and *ORF17* genes, either individually or in combination. Mutant viruses were analyzed for their replication properties in cultured equine dermal cells, PBMC infection efficiency, virus-induced changes in the PBMC proteome, and cytokine and chemokine expression profiles. *ORF1*, *ORF2*, and *ORF17* are not essential for virus replication, but *ORF17* deletion resulted in a significant reduction in plaque size. Deletion of *ORF2* and *ORF17* gene significantly reduced cell-to-cell virus transfer from virus-infected PBMC to EC. EHV-1 infection of PBMC resulted in upregulation of several pathways such as Ras signaling, oxidative phosphorylation, platelet activation and leukocyte transendothelial migration. In contrast, chemokine signaling, RNA degradation and apoptotic pathways were downregulated. Deletion of *ORF1*, *ORF2* and *ORF17* modulated chemokine signaling and MAPK pathways in infected PBMC, which may explain the impairment of virus spread between PBMC and EC. The proteomic results were further confirmed by chemokine assays, which showed that virus infection dramatically reduced the cytokine/chemokine release in infected PBMC. This study uncovers cellular proteins and pathways influenced by EHV-1 after PBMC infection and provide an important resource for EHV-1 pathogenesis. EHV-1-immunomodulatory genes could be potential targets for the development of live attenuated vaccines or therapeutics against virus infection.

## 1. Introduction

Alphaherpesviruses are ubiquitous pathogens affecting human and animal populations [1,2,3,4]. Herpesviruses have evolved an intricate relationship with their hosts [5,6]. Although host immunity is generally successful in controlling infections and minimizing pathogenicity, herpesviruses are successful pathogens and a constant nuisance [7,8]. Herpesviruses employ an array of strategies to evade host immune responses and devote a large number of their genes to block the immune response at multiple levels [9,10]. Gene products of herpesviruses are known to subvert several cellular pathways by virtue of their interaction with host proteins [6,11,12].

Equine herpesvirus type 1 (EHV-1) and type 4 (EHV-4) belong to the genus *Varicellovirus* in the subfamily *Alphaherpesvirinae* and are important pathogens that infect horses [13,14]. EHV-1 predominantly causes upper respiratory tract infection [15]. Following infection and initial replication in the respiratory epithelium, EHV-1 infects mononuclear cells, enters the systemic circulation, and results in cell-associated viremia [16,17]. By virtue of infected peripheral blood mononuclear cells (PBMC), EHV-1 spreads throughout the body [18]. Virus transfer from infected PBMC to endothelial cells (EC) in the gravid uterus and central nervous system, followed by replication in EC, is responsible for the disease outcomes: abortion and neurological disorders, primarily myeloencephalopathy [19,20,21]. Although cell-associated viremia has been reported for other alphaherpesviruses, including pseudorabies (PrV) and varicella zoster virus (VZV), EHV-1 can spread through transient interactions between PBMC and EC without being neutralized by antibodies through a mechanism that requires the coordination of several host and viral proteins [22,23,24,25,26,27].

The viral and host factors responsible for cell-to-cell spread have not yet been fully identified for EHV-1. It was postulated that EC infection is established upon close contact of infected PBMC with EC upon which cell-to-cell virus transfer can take place [28,29]. Factors favoring adhesion of the two cell types and establishing cell-to-cell contact are essential for viral spread [30,31]. In previous studies, we have discovered the essential role of glycoprotein B (gB) and the unique-short region 3 (US3) protein kinase in virus transfer between PBMC and EC [32]. gB facilitates the cell-to-cell spread of EHV-1 by promoting membrane fusion between two adjacent cells [33,34,35], while the unique-short protein kinase pUS3 modulates the actin cytoskeleton and is implicated in adhesion molecule expression [36,37].

We have also identified two immunomodulatory proteins encoded by open reading frame 1 (*ORF1*) and *ORF17*, which are the homologs of the unique-long region 56 (*UL56*) and *UL43* of human herpes simplex virus 1 (HSV-1), respectively [38,39]. The *ORF1* and *ORF17* gene products co-operate to cause major histocompatibility complex I (MHC) down-regulation on the surface of infected PBMC [9,40,41]. Similar mechanisms have been reported for other herpesviruses [42,43,44,45,46]. The *ORF1* and *ORF17* proteins are Golgi-associated transmembrane (TM) proteins that are expressed early during infection, co-localize with each other, and interfere with MHC-I presentation on the cell surface [9]. The *ORF2* gene product is considered a virulence factor for EHV-1 [47]. Previous in vivo studies have revealed the involvement of *ORF1* and *ORF2* in modulating cytokine and chemokine responses in infected horses and subsequently minimizing the course of the disease [48,49,50].

Based on the published literature, we hypothesized that the three-immunomodulatory viral proteins encoded by *ORF1*, *ORF2*, and *ORF17* might play a role in the infection cycle of EHV-1; particularly, virus transfer between PBMC and EC, which is an important step in virus pathogenesis. Identifying viral proteins involved in cell-to-cell virus-spread and determining the underlying mechanisms may provide a potential starting point to develop efficient therapeutics against EHV-1 infection. Therefore, we explored the role of these viral proteins in virus transfer between PBMC and EC and in modulating host cellular pathways. Our most salient findings are that (i) *ORF1*, *ORF2*, and *ORF17* are dispensable for virus replication in equine epithelial cells; (ii) the *ORF2* and *ORF17*, but not *ORF1*, gene products play essential roles in PBMC-EC spread; (iii) the *ORF1*, *ORF2*, and *ORF17* gene products modulate mitogen-activated protein kinase (MAPK) and cytokine and chemokine signaling pathways.

## 2. Materials and Methods 

### 2.1. Cells and Viruses 

Equine dermal (ED) cells (CCLV-RIE 1222, Federal Research Institute for Animal Health, Greifswald, Germany) were propagated in Iscove’s modified Dulbecco’s medium (IMDM; Pan^TM,^ Biotech, Aidenbach, Germany), supplemented with 20% fetal bovine serum (FBS; Biochom^TM^ GmBH, Berlin, Germany), 1 mM sodium pyruvate (Pan^TM^ Biotech, Aidenbach, Germany), 1% nonessential amino acids (NEAA; Biochom^TM^ GmBH, Berlin, Germany), and P-S solution (100 U/mL penicillin: Panreac^TM^, AppliChem GmBH, Darmstadt, Germany; 100 µg/mL streptomycin: Alfa Aesar^TM^, Thermo Fisher Scientific, Kandel, Germany (P-S). Equine EC, isolated from the common carotid artery as described previously [51], and human embryonic kidney cells (293T) were propagated in Dulbecco’s modified Eagle’s medium (DMEM; Biochom^TM^ GmBH, Berlin, Germany), supplemented with 10% FBS and P-S. Equine PBMC were isolated from heparinized blood collected from healthy, EHV-1- and EHV-4-negative horses using Biocoll^®^ (Biochom^TM^ GmBH, Berlin, Germany) as described before [32]. Blood collection was performed according to the rules of Institutional Animal Care and Committee of Berlin (Landesamt für Gesundheit und Sociales, L 0294/13). Separated PBMC were washed in phosphate buffered saline (PBS; 137 mM NaCl, 2.7 mM KCl, 10 mM Na_2_HPO_4_ and 1.8 mM KH_2_PO_4_), re-suspended in RPMI-1640 medium (Pan^TM^ Biotech, Aidenbach, Germany) supplemented with 10% FBS, 2 mM L-glutamine (Pan^TM^ Biotech, Aidenbach, Germany), and 1% P-S.

Mutant viruses were derived from EHV-1 strain Ab4 [52] cloned as an infectious bacterial artificial chromosome (BAC; pAb4). pAb4 contains a mini-F cassette in which the enhanced green fluorescent protein (EGFP) gene is driven by the human cytomegalovirus immediate early promoter [53]. Viruses were reconstituted by transfection of BAC DNA into 293T cells with polyethylenimine (Polysciences^TM^, GmBH, Hirschberg, Germany) [33]. Reconstituted viruses were propagated and titrated in ED cells.

### 2.2. Engineering of EHV-1 BAC Mutants and Revertants 

pAb4 was maintained in *Escherichia coli* GS1783 cells and grown in Luria-Bertani broth (Roth^TM^, Karlsruhe, Germany) supplemented with 30 µg/mL chloramphenicol [40,53]. Two-step Red-mediated recombination (en passant) was used for genetic manipulation of the EHV-1 genome to create mutant viruses [54]. Primers used for the generation of the mutant viruses are given in Table 1. Briefly, to create deletions of *ORF1* and *ORF2*, a fragment flanked by homologous arms for the desired target regions was PCR amplified by using a kanamycin resistance (kan^r^) gene present in plasmid pEP-Kan-S2 [55]. Electrocompetent GS1783 cells were electroporated with the purified PCR product and incubated at 32 °C for 48 hours (h). DNA from kanamycin-resistant bacterial colonies were screened by restriction fragment length polymorphism (RFLP) analysis and compared with the restriction digestion pattern of the parental viral BAC DNA [56,57]. Selected intermediate clones were subjected to the second step of Red-mediated recombination to induce removal of the kan^r^ gene from the BAC after adding 1% L( + )-arabinose (Roth^TM^, Karlsruhe, Germany). Final clones with gene deletions were confirmed by RFLP analysis and specific gene sequencing of the mutation site (LGC® sequencing service, Berlin, Germany). For virus reconstitution, 2 µg BAC DNA were transfected into 293T cells and the reconstituted viruses were subsequently propagated in ED cells. For multiple gene deletions from the same virus, each gene was deleted successively in the BAC before final virus reconstitution. The single gene deleted mutant lacking *ORF 1* and *ORF17* was previously generated and characterized [9,58]. Revertant viruses for the deletion mutants were constructed as described previously [59]. For construction of revertant cassettes, plasmids encoding the target gene with kan^r^ gene were constructed. Briefly, target genes were PCR amplified using primers P11-P12 and P21-P22 for *ORF1* and *ORF2*, respectively. The PCR products were digested with appropriate restriction enzymes (RE) and inserted into pcDNA3 vector (Invitrogen^TM^, Karlsruhe, Germany) resulting in pcDNA3_ORF1 and pcDNA3_ORF2 recombinant plasmids. For construction of pcDNA3_ORF1_kan^r^ and pcDNA3_ORF2_kan^r^, the kan^r^ gene was PCR amplified from pEP-kan-S2 plasmid with P13-P14 and P23-P24 primers, respectively. The resulting PCR products were digested with appropriate restriction enzymes and inserted into pcDNA3_ORF1 and pcDNA3_ORF2. Correct insertion was confirmed by nucleotide sequencing. *ORF17* revertant BAC was constructed by PCR amplification of kan^r^ genes with *ORF17* homologous arm sequences using primers (P5 and P6). The resulting PCR product was used for a two-step Red-mediated recombination as described above.

### 2.3. Growth Kinetics and Plaque Size Assay

The influence of *ORF1*, *ORF2*, and *ORF17* gene deletions on virus replication was evaluated by one-step growth kinetics. EC or ED cells were infected with Ab4 wild-type (Ab4-wt) or mutant viruses (Ab4∆ORF17, Ab4∆ORF1, Ab4∆ORF2, Ab4∆ORF17/ORF2, Ab4∆ORF1/ORF2, Ab4∆ORF1/ORF17, and Ab4∆ORF1/ORF2/ORF17) at a multiplicity of infection (MOI) of 0.1. Virus-infected cells were incubated at 4 °C for 1 h to allow virus attachment and then at 37 °C for 1 h to permit virus entry. After incubation, cells were treated with citrate buffer (40 mM citric acid, 10 mM HCl, and 135 mM NaCl; pH 3.0) for 30 s to inactivate cell-free viruses, and then washed twice with IMDM medium and PBS. Fresh medium was added and cells were incubated at 37 °C under a 5% CO_2_ atmosphere. Supernatant and cell pellet were collected separately at 0, 6, 12, 24, 48, and 72 h post-infection (hpi) to determine cell-free and cell-associated viral titers. Collected samples were titrated on confluent ED cells, overlaid with 1.5% (*w/v*) methylcellulose (Sigma-Aldrich^TM^, Taufkirchen, Germany) in EMEM, and ultimately fixed with 4% paraformaldehyde in PBS at 72 hpi. Fixed cells were stained with 0.1% (*w/v*) crystal violet solution in PBS, plaques were counted, and results were expressed as plaque-forming units (PFU) per milliliter (mL). Data are presented from three independent and blinded experiments. To determine cell-to-cell spread, plaques induced by Ab4-wt and mutant viruses were measured at 48 hpi on ED cells. Virus plaques were imaged using a Zeiss Axiovert.A1 fluorescent microscope, equipped with an Axiocam 503 camera (Carl Zeiss AG, Jena, Germany). In total, 150 plaque images were processed for each virus and actual plaque diameters were measured using ImageJ^®^ software (National Institute of Health, Bethesda, MD, USA).

### 2.4. Co-Cultivation Assay

To evaluate EHV-1 transfer from infected PBMC to EC, a co-cultivation assay was performed as described previously [32,60]. EC were grown to confluency on collagen IV-coated 24-well plates (BioCoat^TM^, Glendale, AZ, USA). PBMC were infected with either Ab4-wt or mutant viruses at MOI of 0.1 for 24 h. Virus-infected PBMC were sorted for EGFP expression by fluorescent-activated cell sorting (FACS) using FACSAria (BD Bioscience^TM^, Heidelberg, Germany) (Supplementary Figure S1). Sorted PBMC (2 × 10^4^) were treated with citrate buffer as described above and overlaid on an EC monolayer in the presence of EHV-1 neutralizing antibodies (final EHV-1 antibody titer of 1:2048) for 2 h (“contact”). Alternatively, infected PBMC were placed into a 24-well transwell TC-Inserts (0.4 µm pore size; Sarstedt^TM^, Nümbrecht, Germany) in the presence of EHV-1-neutralizing antibodies without direct contact with EC (“no contact”). The “no contact” controls were used to assess the efficiency of citrate buffer treatment (inactivation of cell-free virus) and EHV-1- neutralizing antibodies and to ensure that there was no infection with free viruses. After incubation, PBMC were washed with PBS, and EC were overlaid with 1.5% methylcellulose medium. The plates were incubated for 24 h and EGFP-positive plaques on the EC monolayer were counted using an inverted Zeiss Axio Vert.A1 fluorescent microscope. The co-cultivation assay was performed with Ab4-wt and mutant viruses. The number of virus plaques produced by Ab4-wt infected PBMC was compared with mutant viruses. Results were interpreted from three independent blinded replicates.

### 2.5. Flow Chamber Assay

The flow chamber assay was performed to evaluate virus transfer from infected PBMC to the EC under dynamic “flow” conditions as described earlier [32]. EC were grown to confluency in collagen IV-coated flow chamber µ-slides (Ibidi^®^ GmBH, Gräfelfing, Germany). PBMC were infected with either Ab4-wt or mutant viruses at an MOI of 0.1 for 24 h. Virus-infected PBMC were FACS-sorted as described above. Sorted PBMC (2 × 10^4^) were treated with citrate buffer and re-suspended in DMEM containing EHV-1- neutralizing antibodies. EC containing flow chamber slides were connected to a perfusion system (Multi-Syringe Pump, World precision instruments, Friedberg, Germany) and PBMC were allowed to flow over the EC at the physiological flow rate for mammalians (0.5 mm/s) at 37 °C [61]. PBMC-containing medium, flowing though the chamber slide, was collected as waste on the other side. Following PBMC flow, EC were washed and incubated for 24 h to allow virus transfer and the development of viral plaques. EGFP-positive plaques on the EC monolayer were counted (Zeiss Axio Vert.A1). The “no contact” setup was always included for each experiment. The number of plaques counted for Ab4-wt and mutant viruses were compared to evaluate the efficiency of virus transfer between PBMC and EC under flow conditions. The experiment was performed independently in a blinded fashion three times.

### 2.6. Infection of Equine PBMC Subpopulations

To determine which PBMC subpopulation (i.e., T lymphocytes, B lymphocytes, or monocytes) is responsible for virus transfer to EC, each subpopulation was sorted, and virus spread was assessed by the flow chamber assay. Briefly, PBMC were labeled with (1:200 diluted) primary mouse monoclonal antibodies against equine CD3 (T lymphocyte), IgM (B lymphocyte) and CD14 (monocyte). Antibodies were kindly provided by Dr. Bettina Wagner, Cornell University, Ithaca, NY, USA. Stained PBMC were labeled with secondary Alexa Fluor 488-conjugated goat anti-mouse IgG antibody (Invitrogen^TM^, Karlsruhe, Germany) and sorted. Sorted populations (T-, B-lymphocytes, monocytes; 2 × 10^4^) were infected with Ab4-wt or mutant viruses at an MOI of 0.1 for 24 h at 37 °C. After incubation, cells were treated with citrate buffer, resuspended in medium containing EHV-1-neutralizing antibodies, and the flow chamber experiment was performed as described above. Virus transfer between each PBMC subpopulation and EC was evaluated by counting the number of viral plaques at 24 hpi and compared between Ab4-wt and mutant viruses. The experiment was performed independently in a blinded fashion three times.

### 2.7. Equine Epithelial Cell-PBMC Contact Assay 

The equine epithelial cell-PBMC contact assay was performed to assess virus transfer from epithelial cells to PBMC, and subsequently from PBMC to EC as described previously with modifications [62]. Briefly, ED cells were seeded into a 3-µm pore size 24-well transwell insert (Sarstedt^TM^, Nümbrecht, Germany) and incubated at 37 °C for 24 h. The cells were inoculated with Ab4-wt or mutant viruses at an MOI of 0.1 and incubated for 1 h. Infected cells were washed twice, treated with citrate buffer, and supplemented with growth medium containing EHV-1-neutralizing antibodies. The transwell insert was inverted, and 1 × 10^5^ PBMC resuspended in medium containing virus-neutralizing antibodies were added and incubated at 37 °C for 24 h. The virus transfer from epithelial cells to PBMC in the presence of neutralizing antibody was assessed by counting the number of EGFP-positive (EHV-1-infected) PBMC after 24 h using a Zeiss Axiovert.A1 fluorescent microscope. After counting, infected PBMC were applied to confluent EC in a flow chamber setup. The experiment was performed independently in a blinded fashion three times.

### 2.8. Whole-Cell Proteomic Analysis

#### 2.8.1. Sample Preparation

Label-free quantitative proteomic analysis was performed to determine differentially expressed proteins in PBMC infected with EHV-1. PBMC were infected with Ab4-wt or mutant viruses at an MOI of 1. At 24 hpi, 10^5^ PBMC were FACS-sorted. For mock-infected PBMC controls, T- and B-lymphocytes, as well as monocytes, were mixed at percentages comparable to the populations of infected PBMC (T- and B-lymphocytes, as well as monocytes, were mixed at a ratio of 21:13:66). After sorting, PBMC were washed twice with PBS and stored at −80 °C until further processed. This process was repeated four independent times and we ended up with 4 batches of infected PBMC.

For protein digestion and LC/MS analysis, the cell pellet of each batch was lysed in lysis buffer (4% SDS in 50 mM triethylammonium bicarbonate buffer, pH 8.5, supplemented with 25 units Benzonase nuclease (Merck) and 1x protease inhibitor EDTA free). Protein reduction and alkylation were accomplished using 5 mM DTT for 30 min at 55 °C and 40 mM CAA for 30 min at room temperature in the dark, respectively. Protein digestion and peptide clean-up was performed using the single-pot, solid-phase-enhanced sample-preparation (SP3) technology [60,63]. Protein digestion was accomplished with LysC and trypsin, both in 1:50 ratio at 37 °C overnight. LC/MS analysis was performed using an UltiMate 3000 RSLC nano LC system coupled on-line to an Orbitrap Elite mass spectrometer (Thermo Fisher, Waltham, MA, USA). Reversed-phase separation was performed using a 50 cm analytical column (in-house packed with Poroshell 120 EC-C18, 2.7 µm, Agilent Technologies, Santa Clara, CA, USA).

For LC/MS data analysis, raw data were processed using MaxQuant software (version 1.6.1.0; Max Planck Institute of Biochemistry, Planegg, Germany) [64] with default settings. MS2 spectra were searched against horse or human databases supplemented with the protein sequence of EHV-1 Enzyme specificity was set to trypsin. Cysteine carbamidomethylation was included as a fixed modification, and methionine oxidation was used as a variable modification. The mass tolerances were set to 10 ppm for the first search and 4.5 ppm for the main search for MS while 0.6 Da was used for MS2. Global false discovery rates for peptide and protein identification were set to 1%. The match-between-runs and label-free quantification options were enabled.

#### 2.8.2. Data Analysis and Interpretation 

PBMC protein expression data were obtained using the LFQ approach from MaxQuant. The data was used to create a profile of expression levels with Perseus software (version 1.6.1.3; Max Planck Institute of Biochemistry, Planegg, Germany) [65]. LFQ values were log_2_-transformed and missing values were imputed. Student’s two-sample *t*-test was used to assess statistical significance of differentially expressed protein abundances using a 1% permutation-based FDR (*q*-values) correction for multiple hypotheses testing with Perseus software [66]. Proteins that showed a fold-change of at least 1.5 (*p* < 0.05) were considered differentially expressed. Differentially expressed proteins were mapped to the gene ontology (GO) database, and the number of proteins at each GO term was computed. The results from label-free proteomics were used as the target list. GO and Kyoto Encyclopedia of Genes and Genomes (KEGG) annotation for each protein in the search database were retrieved from GO (http://www.geneontology.org/) and KEGG Pathway database (http://www.genome.jp/Pathway), respectively. KEGG pathway enrichment analysis of the correlation was carried out using *p* values adjusted with Benjamini correction for false discovery rate. In the GO enrichment analysis, proteins were classified by GO annotation into three categories: biological process, cellular compartment, and molecular function. For each category, a two-tailed Fisher’s exact test was employed to compare the enrichment of the differentially expressed protein against all identified proteins, and corrected *p*-values < 0.05 were considered significant [67,68].

### 2.9. Multiplex Equine Cytokine Assay

Cytokines and chemokines released by control or EHV-1-infected PBMC were quantified using Milliplex^®^ MAP equine cytokine/chemokine magnetic bead-based Multiplex kit. The kits enable simultaneous analysis of 23 cytokine and chemokine biomarkers (EMD Millipore, Billerica, MA, USA) [69]. PBMC (1 × 10^6^) were infected with Ab4-wt, mutant viruses at MOI of 1. Supernatants from control, Ab4-wt/mutant (Ab4∆ORF17, Ab4∆ORF1, Ab4∆ORF2, Ab4∆ORF17/ORF2, Ab4∆ORF1/ORF2, Ab4∆ORF1/ORF17, and Ab4∆ORF1/ORF2/ORF17) infected PBMC was collected at 3, 6, and 24 hpi. In another experiment, 1 × 10^6^ PBMC were infected with Ab4-wt at MOI of 1. At 24 hpi, infected PBMC were applied over EC in a 24-well plate and incubated for 6 h. Supernatants were collected at 3 and 6 h, and stored at −80 °C until used. Cytokine and chemokine quantification in the supernatant (both PBMC and PBMC-EC) was performed as per the manufacturer’s instructions using the Milliplex^®^ MAP equine cytokine/chemokine magnetic bead based Multiplex kit with Luminex-based detection system (Luminexcorp^TM^ MAGPIX^®^ system, Austin, TX, USA) with xPONENT^®^ software (Luminexcorp^TM^, Austin, TX, USA). All samples were run in duplicate.

### 2.10. Statistical Analysis 

Statistical analyses were performed using GraphPad PRISM^®^ 5.01 software (San Diego, CA, USA). Normally distributed group samples were analyzed with a one-way ANOVA test followed by a multiple comparisons test. For all analyses, a ‘*p*’ value of less than 0.05 was considered significant.

## 3. Results

### 3.1. The ORF1, ORF2 and ORF17 Genes are Dispensable of EHV-1 Replication

**Figure 1:**
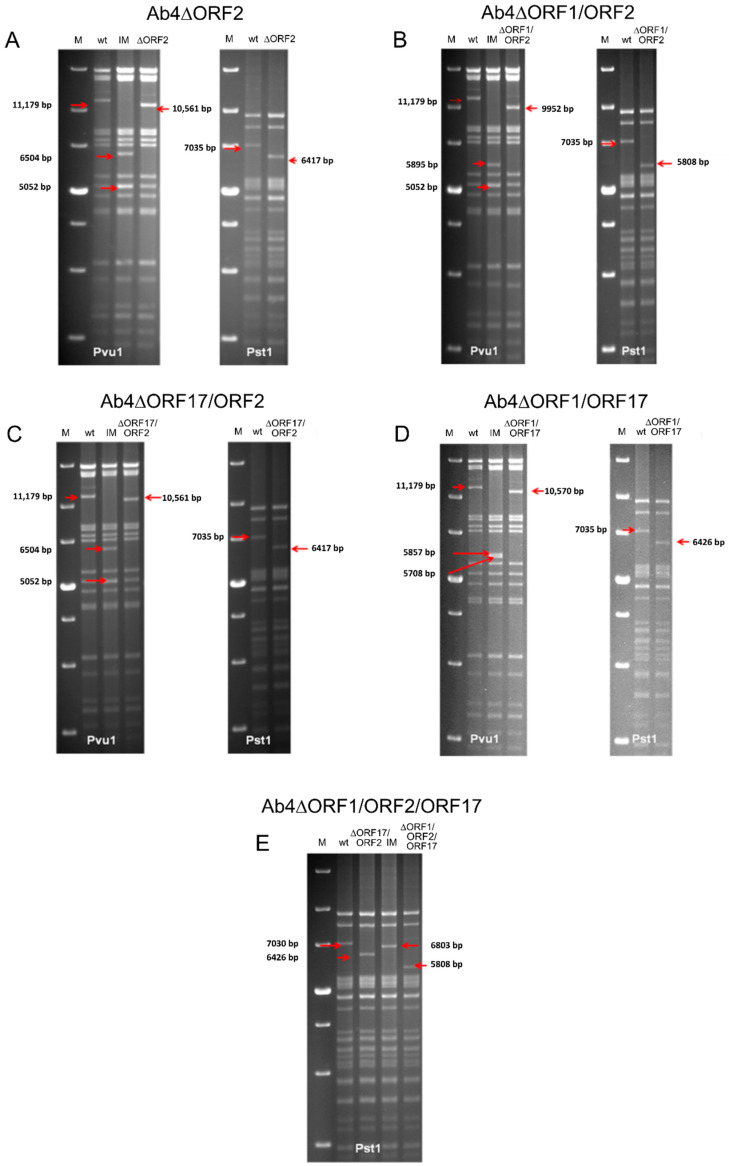
Restriction fragment length polymorphism (RFLP) analysis for characterization of constructed BAC mutants. Purified DNAs from Ab4-wt, intermediate clone with kanamycin cassette and final clone with gene deletion BAC were digested with *Pst1* and *Pvu1*. Fragments in the intermediate and final mutant clones that appeared as a result of the deletion of sequence were marked by red arrows. Construction of (**A**) Ab4∆ORF2, (**B**) Ab4∆ORF1/ORF2, (**C**) Ab4∆ORF17/ORF2, (**D**) Ab4∆ORF1/ORF17 and (**E**) Ab4∆ORF1/ORF2/ORF17 BAC mutants. M—1 kb plus DNA ladder (Thermo Fischer Scientific, Paisley, UK), wt—EHV-1 Ab4 strain wildtype virus, IM—intermediate BAC clone with kanamycin cassette insertion, ∆—specific gene deletion and arrow—band shifts.

EHV-1 Ab4 mutants lacking *ORF1*, *ORF2*, and *ORF17* genes as single, double, or triple gene deletions were successfully generated using *en passant* mutagenesis. Specific gene deletions made in the current study were confirmed by sequencing and RFLP (Figure 1A–E), and all mutant viruses (Ab4∆ORF17, Ab4∆ORF1, Ab4∆ORF2, Ab4∆ORF17/ORF2, Ab4∆ORF1/ORF2, Ab4∆ORF1/ORF17, and Ab4∆ORF1/ORF2/ORF17) were successfully reconstituted.

**Figure 2:**
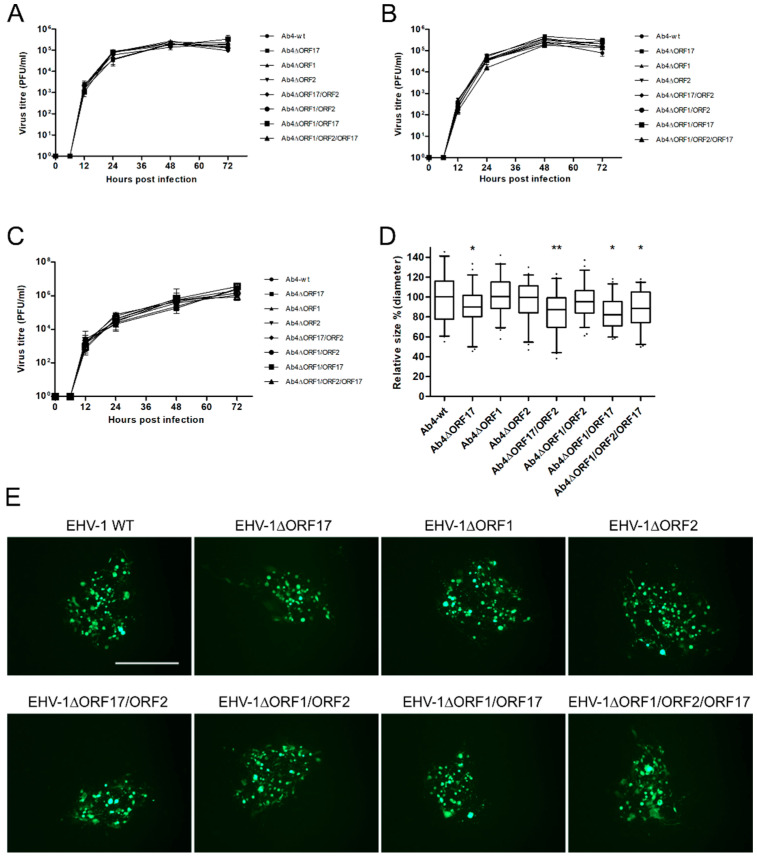
Characterization of mutant viruses. Growth kinetics of mutant Ab4 viruses on ED cells infected at an MOI of 0.1. (**A**) Infected cells and (**B**) supernatant were collected, and virus titers were determined at different time points. (**C**) For EC cells, both cells and supernatants were collected together at each time point and the virus titer was determined. Mean virus titers ± standard deviation are given. (**D**) Mean diameters of 150 plaques were measured for each virus. The plaque diameter of Ab4-wt was set to 100% and the mean diameter ± standard deviation is given; (*n* = 3; One-way ANOVA test followed by multiple comparisons test). *—*p* < 0.05, **—*p* < 0.01. (**E**) Representative images of plaques induced by the virus mutants on ED cells. Scale bar = 500 µm.

Three independent growth kinetic experiments were performed to evaluate the replicative potential of all mutant viruses in ED and EC cells. The data revealed that all mutant viruses replicated at levels that were virtually identical to those of Ab4-wt (Figure 2A–C). Plaque size assays were performed to assess cell-to-cell virus spread in ED cells (Figure 2D,E). Among the different gene deletion mutants, deletion of *ORF17* significantly reduced plaque sizes compared to Ab4-wt (*p* < 0.05). Similarly, deletion of *ORF17* in combination with other mutations (Ab4∆ORF1/ORF17, Ab4∆ORF17/ORF2, and Ab4∆/ORF1/ORF2/ORF17) also resulted in similar reductions of plaque sizes (13–17%).

### 3.2. ORF2 and ORF17 Genes Are Important for Transfer to EC

**Figure 3:**
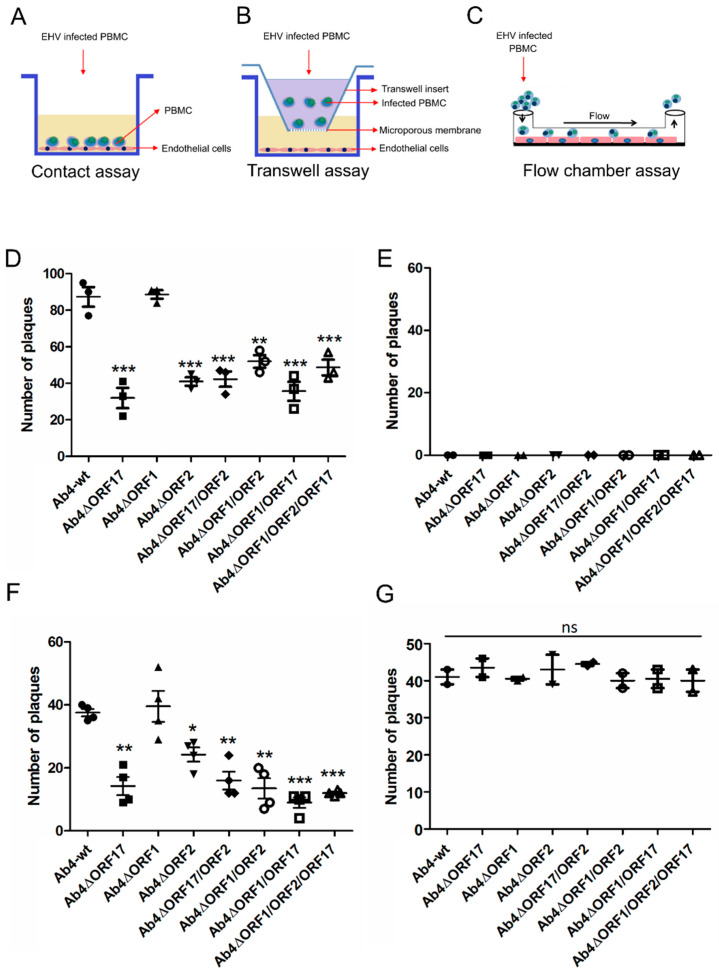
Virus transfer from EHV-1-infected PBMC to EC under static and dynamic flow conditions. Schematic diagram of (**A**) the contact assay, (**B**) the transwell assay, and (**C**) the flow chamber assay. PBMC were infected with Ab4-wt or mutant viruses at an MOI of 0.1 for 24 h. (**D**) The contact assay was performed after sorting of infected cells. Virus plaques were counted after 24 h. (**E**) As a control, infected PBMC were placed into a transwell insert without direct contact between PBMC and EC. The flow chamber assay was performed for the mutant (**F**) and corresponding revertant (**G**) viruses. Data are presented as mean plaque numbers ± SD; (*n* = 3; One-way ANOVA test followed by multiple comparisons test). *—*p* < 0.05, **—*p* < 0.01, ***—*p* < 0.001. ns—not significant.

Co-cultivation (Figure 3A) and flow chamber (Figure 3C) assays were performed to evaluate virus transfer between virus-infected PBMC and EC under static and dynamic conditions, respectively. Ab4-wt-infected PBMC were able to transfer the virus to EC in the presence of neutralizing antibodies under both static and flow conditions as described previously [32]. No virus transfer was observed in the “no contact” transwell setup, where infected PBMC were physically separated from EC (Figure 3B,E). Among the single-gene deletion mutant viruses, the *ORF2*- and *ORF17*-negative Ab4 mutants exhibited significantly (*p* < 0.05) reduced virus spread to EC under static conditions (co-cultivation) as evidenced by reduced plaque numbers. Deletion of *ORF2* and *ORF17* genes resulted in 65% and 52% reduction of virus transfer to EC, respectively (Figure 3D). In addition, deletions of two or three genes in the same virus (double and triple gene deletion mutants) also resulted in a 42–65% reduction in virus transfer (Figure 3D). No significant difference in the rate of virus transfer was seen for Ab4∆ORF1 when compared to Ab4-wt (*p* > 0.05; Figure 3D). The results of the flow chamber assay mimicked those of the co-cultivation assay in terms of virus transfer events observed for mutant viruses. Deletion of *ORF17* and *ORF2* genes led to a reduction in virus transfer of 65% and 40%, respectively (Figure 3F). Deletion of two genes in combination resulted in a 60–78% reduction in virus transfer (Figure 3F). The Ab4∆ORF1/ORF2/ORF17 triple mutant exhibited a 76% reduction in virus transfer (Figure 3F). Ab4∆ORF1, on the other hand, did not show a significant difference compared to the parental virus.

All revertant viruses (Ab4ORF17R, Ab4ORF1R, Ab4ORF2R, Ab4ORF17R/ORF2R, Ab4ORF1R/ORF2R, Ab4ORF1R/ORF17R, and Ab4ORF1R/ORF2R/ORF17R) were reconstituted for each corresponding deletion mutant and flow chamber assays were performed. The revertant viruses spread from infected PBMC to EC at rates similar to those of parental virus (Figure 3G). In all cases, no virus transfer was seen between infected PBMC and EC under “no contact” conditions (Figure 3E).

### 3.3. Virus Transfer to EC through Different PBMC Subpopulations

**Figure 4:**
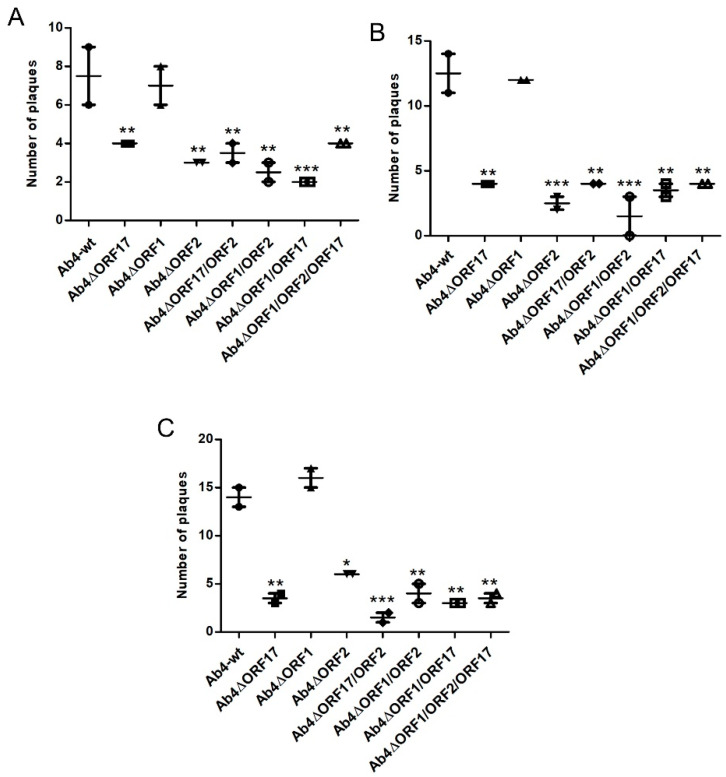
Virus transfer from EHV-1-infected PBMC subpopulations to endothelial cells under dynamic flow condition. PBMC were infected with Ab4-wt or mutant viruses at an MOI of 0.1 for 24 h. Infected PBMC were FACS-sorted and flow chamber assays were was performed for (**A**) T-lymphocytes, (**B**) B-lymphocytes, and (**C**) monocytes. Virus plaques were counted at 24 hpi. The data represent the mean of two independent experiments ± SD. For statistical analysis, one-way ANOVA followed by correction for multiple comparisons test were performed; *—*p* < 0.05, **—*p* < 0.01, ***—*p* < 0.001.

We isolated B-cells, T-cells, and monocytes from whole PBMC by FACS sorting using population-specific antibodies. Cells were then infected with Ab4-wt and mutant viruses, and flow chamber assay was performed. Virus infection assay in PBMC showed that B- and T-lymphocytes as well as monocytes could be infected with Ab4-wt (Table 2) [32]. However, the subpopulation that is mainly responsible for virus transfer to EC is still not clearly established. To address this question, we performed flow chamber assays for each infected PBMC subpopulation, which revealed that all three subpopulations were able to transfer the virus to EC (Figure 4A–C). Similar to the whole PBMC population, PBMC subpopulations infected with individual mutant viruses showed significant reductions in virus transfer to EC (Figure 4A–C), while mutants with double and triple gene deletions showed up to 45–80% reduction in virus transfer. As seen earlier, virus transfer to EC remained unaltered in the case of the Ab4∆ORF1 mutant.

### 3.4. Mimicking the In Vivo Pathway of Virus Spread from Epithelial Cells to PBMC and from PBMC to EC

**Figure 5:**
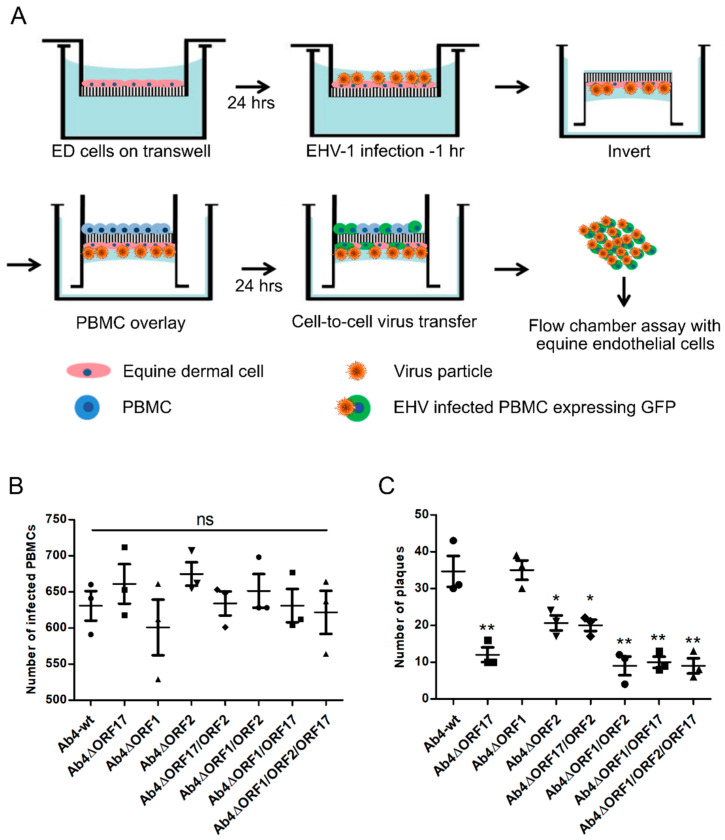
EHV-1 spread between epithelium, PBMC, and EC. (**A**) ED cells were seeded into 3-µm pore size 24-well transwell inserts and incubated at 37 °C for 24 h. Subsequently, ED cells were infected with Ab4-wt or mutant viruses at an MOI of 0.1 for 1 h and treated with citrate buffer. Upon infection, transwell insert was inverted, and 1 × 10^5^ PBMC resuspended in medium containing virus-neutralizing antibodies were added. (**B**) Epithelium-to-PBMC virus transfer was assessed by counting the number of EGFP-positive cells after 24 h. (**C**) Subsequently, infected PBMC were used for flow chamber assay. The data represent means ± SD of three independent experiments. One-way ANOVA was done followed by correction for multiple comparisons; *—*p* < 0.05, **—*p* < 0.01. ns—not significant.

Following inhalation of EHV-1, the virus primarily replicates in respiratory tract epithelial cells, subsequently passes through the basement membrane, establishes cell-associated viremia, and, finally, is transferred to the endothelial epithelium [70]. To mimic EHV-1 pathogenesis in vitro and investigate virus transfer from infected epithelial cells to PBMC and subsequently from PBMC to EC we developed equine epithelial cell-PBMC contact assay (Figure 5A). ED cells grown on a transwell membrane were infected with Ab4-wt or mutant viruses, PBMC suspended in medium containing EHV-1-neutralizing antibodies were added to the inverted transwell to establish contact with ED cells. Twenty-four hours after contact, PBMC were collected and analyzed for the rate of infection by counting EGFP-positive cells. No significant differences in the rate of infection of PBMC were seen between parental and mutant viruses (Figure 5B). In contrast, when we continued the flow chamber experiment with the same infected PBMC, virus transfer to EC was greatly reduced ranging from 35% to 65% for the Ab4∆ORF2 and Ab4∆ORF17 viruses (Figure 5C). Deletion of *ORF1* did not affect virus transfer at any stage.

### 3.5. Comparative Proteomic Analysis

**Figure 6:**
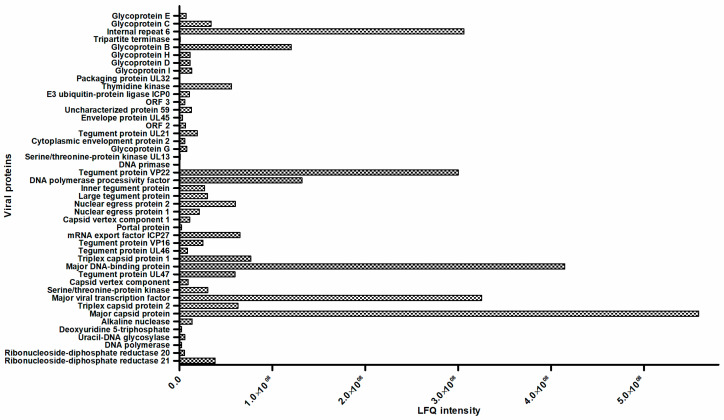
Quantification of viral proteins in EHV-1-infected PBMC at 24 hpi. Absolute quantification of viral proteins quantification in terms of LFQ values are given on the X-axis and the names of the viral proteins are given on the Y-axis; (*n* = 4).

Four replicates of PBMC were prepared from the blood of two horses and each replicate of cells was infected with Ab4-wt or mutant viruses at an MOI of 1. At 24 hpi, infected cells were FACS-sorted and protein extracts were prepared from infected and mock-infected PBMC. Upon analysis, 45 viral proteins (Table 3) and 1300 equine cellular proteins were detected and quantified. Among the quantified host proteins, 141 proteins displayed significant differences in expression levels between Ab4-wt-infected and uninfected PBMCs as identified by at least two high confidence (95%) peptides with *p*-values ≤0.05, as calculated by Perseus software. In total, 63 cellular proteins were upregulated and 78 proteins were downregulated. KEGG-pathway enrichment analysis of differentially expressed proteins (*p* ≤ 0.05) was calculated using Benjamini-corrected Fisher’s exact test. In Ab4-wt-infected PBMC, upregulation of several pathways including platelet activation, Ras signaling, leukocyte transendothelial migration, endocytosis, lysosome, oxidative phosphorylation pathways, and cAMP signaling pathways was detected. On the other hand, downregulation of proteins associated with herpesvirus infection, chemokine signaling, spliceosome, RNA degradation, and apoptotic pathways was observed (Table 4 and Supplementary Table S1). As mentioned above, in total 45 (18 non-structural, 24 structural, and 3 uncharacterized) viral proteins were detected in infected PBMC out of the 78 ORF-encoded proteins by EHV-1 including structural and non-structural viral proteins (Figure 6, Table 3, and Supplementary Table S2). Among the viral proteins in infected PBMC, major capsid protein, major DNA-binding protein, major viral transcription factor, internal repeat 6, and tegument protein VP22 were the most abundant. Among glycoproteins, glycoprotein B and glycoprotein C were most abundant in EHV-1-infected PBMC. There were some differences in the level of expression of certain viral proteins (DNA primase, serine/threonine-protein kinase UL13, ORF protein 3, and gD) in PBMC infected with Ab4-wt and the mutant viruses in proteomic analysis (Supplementary Table S3). Future studies are required to investigate the kinetics of viral proteins in infected PBMC at different time points and their possible role in virus transfer between PBMC and EC. 

While comparing the proteome of PBMC infected with Ab4-wt or mutant viruses, differential expression of more than 100 proteins was observed. Details of pathways differentially modulated are shown in Table 4. Infection with Ab4∆ORF17 resulted in upregulation of proteins associated with herpesvirus infection (lymphocyte function-associated antigen 3 [LFA-3], TNFRSF1A associated via death domain [TRADD], recombination signal binding protein for immunoglobulin kappa J region [RBPJ] I, RBPJ like, Proteasome 26S subunit, non-ATPase 12) and the Fc epsilon receptor I signaling pathways. On the other hand, Ab4∆ORF17 downregulated proteins associated with chemokine signaling (MAPK kinase 1, Ras-related protein Rap-1A, stress-induced phosphoprotein 1) and MAPK (Ras-related protein Rap-1A, Interleukin 1 alpha, Ribosomal protein S6 kinase A5) signaling pathways. It is important to note that proteins such as LFA-3, RPBJ, RPBJL (herpesvirus infection pathways) are involved in cytokine release, leukocyte migration, cell-to-cell adhesion, and signaling receptor pathways. Ab4∆ORF1 infection resulted in upregulation of mTOR signaling, focal adhesion, and chemokine signaling pathway. Ab4∆ORF2 infection resulted in upregulation of herpesvirus infection and chemokine signaling pathways, and downregulation of MAPK signaling pathways. Finally, the triple gene deletion mutant caused upregulation of herpesvirus infection, T-cell signaling, and chemokine signaling pathways and downregulation of MAPK signaling and oxidative phosphorylation pathways. (Supplementary Table S1). From the results, we concluded that the three viral genes modulate several host pathways in addition to the release and regulation of cytokines and chemokines from infected PBMCs.

### 3.6. EHV-1 Infection Modulates Cytokine and Chemokine Profiles of PBMC

**Figure 7:**
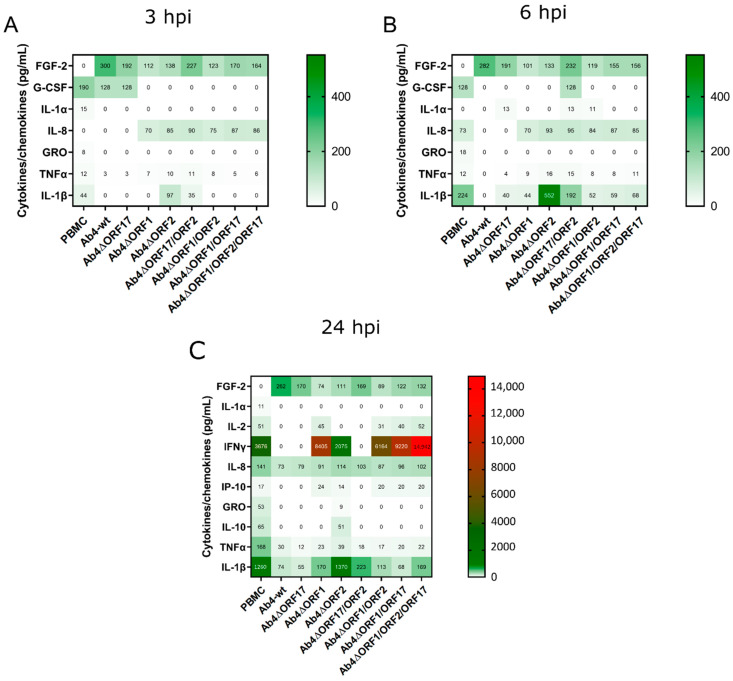
PBMC cytokine and chemokine profile at 3, 6, and 24 hpi. Equine PBMC were infected with either Ab4-wt or mutant viruses at an MOI of 1. (**A**) At 3 hpi, (**B**) 6 hpi, and (**C**) 24 hpi, supernatants were collected, and cytokines and chemokines were quantified using a Milliplex^®^ MAP equine cytokine/chemokine magnetic bead-based Multiplex kit with Luminex-based detection system. Mean concentrations of cytokines and chemokines are given in picogram (pg) per mL; (*n* = 2). hpi—hours post infection.

Equine PBMC were infected with Ab4-wt or mutant viruses at an MOI of 1. Supernatants from infected PBMC were collected at 3, 6, and 24 hpi, and the secreted cytokines and chemokines were quantified using Milliplex^®^ MAP equine cytokine/chemokine magnetic bead-based Multiplex kit with Luminex-based detection system. We detected 12 cytokines in total expressed by PBMCs (naïve and/or EHV-1-infected) out of 23 cytokines tested in the panel. All standards and quality controls were within the specified range. In general, the release of most cytokines and chemokines was strongly inhibited upon Ab4-wt infection of PBMC. Only FGF-2 was expressed in infected PBMC at 6 hpi (Figure 7A,B; Table 5). In contrast, infecting PBMC with Ab4 mutant viruses resulted in the partial or complete restoration of the expression of cytokines and chemokine as evidenced by the expression of G-CSF, IL-1α, IL-1β, IL-8, and TNFα.

At 24 h, naïve PBMC released nine cytokines and chemokines. In contrast, PBMC infected with Ab4-wt or Ab4∆ORF17 released only four cytokines and chemokines (FGF-2, IL-8, TNFα, and IL-1β) (Figure 7C; Table 5). Infection of PBMC with Ab4∆ORF1 or Ab4∆ORF2 restored the release of several cytokines. In the case of the *ORF2* single deletion mutant cytokines including IL-10, IL-8, and IL-1β were released in higher quantities. However, the deletion of *ORF17* in combination with *ORF2* reduced the expression of cytokines (Table 5). All mutants with an *ORF1* deletion resulted in the expression of more cytokines (Figure 7C). IFNγ levels were higher in all *ORF1* deletion mutants. It is worth mentioning that FGF-2 expression was only observed in infected PBMC and that its concentration was always high in the case of Ab4-wt, followed by *ORF17/ORF2* and *ORF17* deletion mutants at different time points.

**Figure 8:**
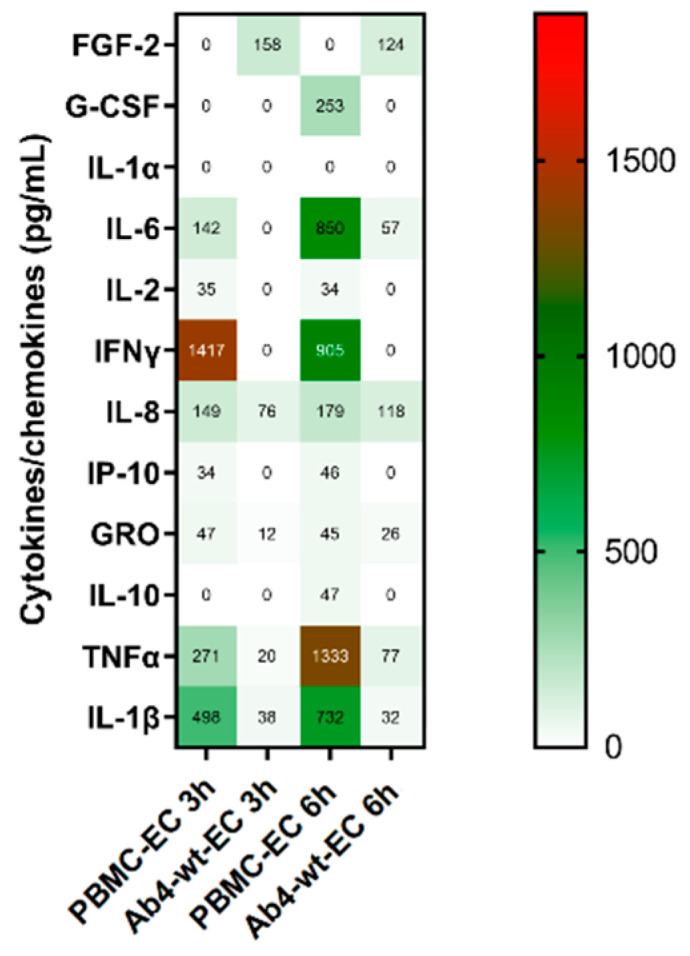
PBMC cytokine and chemokine profile of PBMC-EC co-culture at 3 and 6 h. Equine PBMC were infected with Ab4-wt at an MOI of 1. Infected PBMC were collected and co-cultivated with EC, incubated for 3 and 6 h, supernatants were collected, and cytokines/chemokines concentrations were measured using Milliplex^®^ MAP equine cytokine/chemokine magnetic bead-based Multiplex kit with Luminex-based detection system. Mean concentration of cytokines and chemokines were given in pg per mL; (*n* = 2).

We were further interested in studying the cytokine release profile of EHV-infected PBMC in the presence of EC. PBMC (1 × 10^6^) were infected with Ab4-wt at an MOI of 1. At 24 hpi, infected PBMC were applied over EC, and supernatants were collected at 3 and 6 h for cytokine estimation. In PBMC-EC co-cultures, non-infected PBMC released more cytokines than virus-infected PBMC. Further, Ab4-wt infected PBMC in the presence of EC did not show much difference in terms of release of additional cytokines and chemokines, except release of IL-6 (Figure 8). Overview of cytokines produced, cell source, targets, and functions are given in Supplementary Table S4.

Taken together, infection of PBMCs with Ab4-wt mostly resulted in downregulation of cytokine expression and deletion of different viral genes (*ORF1*, *ORF2*, and *ORF17*) clearly altered the cytokine expression profile and restored cytokine expression partially at different time points. *ORF2* gene deletion expressed higher levels of IL-1β cytokine at all time points.

## 4. Discussion

EHV-1 infection of PBMC is a critical step in deciding EHV-1 pathogenesis; thereby the virus can spread from respiratory epithelium to the endothelium without being captured by the host immune system. As described earlier, the process of virus transfer is complex, requires coordinated action of viral proteins, adhesion molecules expressed by both cells, and cytokines and chemokines for facilitating inter-cellular adhesion, intra-cellular trafficking, and cellular polarity [71,72]. Several viral proteins are involved in mediating such processes. Our previous studies revealed that gD, gB, and US3 proteins play an essential role in PBMC and EC infection as well as virus transfer between the two compartments [32,59]. Cell-to-cell virus transfer is considered to be a mechanism of immune evasion and immunomodulation properties of herpesviruses [73]. Earlier studies revealed that EHV-1 can evade host immune response by modulating MHC-I expression on the surface of the infected cells [74] with the help of the viral proteins UL49.5, ORF1, and ORF17 [9,38,40,41,75]. Further, *ORF1* and *ORF2* deletion mutants showed significantly reduced virus shedding, a shorter course of pyrexia, and modulated cytokine response with attenuation of virulence in comparison to Ab4-wt in in vivo studies in ponies [50]. Experimental infection with *ORF1* and *ORF71* deletion mutants revealed a brief period of pyrexia, low virus shedding, and decreased cytokine response (IFNα, IL-10, and soluble CD14); however, they had comparable viremia to Ab4-wt [48]. In another study, *ORF2* deletion mutant was attenuated; however, it had strong immunogenicity without altering viremia in Icelandic horses [49,76]. Failure to induce T-cell response was suggested as a reason for viremia in both Ab4-wt and mutant viruses infected horses [48,49,50]. As animal experiments were performed in non-pregnant horses and no distinct neurological signs were observed following infection with Ab4-wt and mutant viruses, it is unclear that cell-associated viremia with mutant viruses can result in subsequent endothelial infection or not. With this background, we hypothesized that EHV-1 immunomodulating proteins (*ORF1*, *ORF2*, and *ORF17*) are essential for virus pathogenesis and play an important role in virus spread from PBMC to EC.

*ORF1*, *ORF2*, and *ORF17* genes of EHV-1 are dispensable for virus replication in equine cells as confirmed by growth kinetics and plaques size assays. Double and triple gene deletion mutants also replicated normally as with the parental virus. Only *ORF17* deletion mutant showed a significant, but slight, reduction in plaque size, ranging between 10–15%. Albeit the plaque size reductions were less, similar plaque size reductions of UL43 (*ORF17* homolog) deletion mutants were observed in HSV-1 and PrV [77,78]. Despite the non-essential character, the conservation of UL43 homologs function in relation to plaque size within the alphaherpesviruses is interesting.

We show here that PBMC can be infected, to similar levels, with all mutant viruses without any significant differences. Co-cultivation and flow chamber assays with parental EHV-1 virus showed efficient virus spread from infected PBMC to EC as reported previously [32]. However, deletion of *ORF17* and *ORF2* as single-gene deletion mutant significantly reduced virus transfer between PBMC and EC under static and flow conditions. Furthermore, *ORF17* and *ORF2* mutants (as double and triple gene deletions mutants in combination with *ORF1*) also reduced virus transfer to EC. Double and triple gene deletions showed an additive reduction in virus transfer to EC in a flow condition compared to static condition, which can be attributed to a synergetic effect on the functional dynamic rolling of infected PBMC over EC. The flow chamber system creates homogenous fluid shear stress in the endothelium similar to that observed in the blood vessel environment in vivo. Fluid flow over the endothelium results in ranges of ion fluxes, modulation of several pathways including biochemical pathways and gene and protein expression in both in vitro and in vivo. Specifically, shear forces modulate cell surface transmembrane adhesion molecules via cytoskeleton by integrin-dependent activation of MAP kinases via Ras GTPase and RhoA activation [79]. Furthermore, the rolling of PBMC over the endothelium provides kinetics of cell-to-cell adhesion and increases the binding strength of the interaction between the two cells [80], which was the advantage of the flow chamber system rather than the static condition. As will be discussed later, *ORF2* and *ORF17* modulated the MAP kinase and Ras GTPase pathways, which might explain the reduction of virus spread.

In the epithelium-PBMC contact assay, all mutant viruses transferred from infected epithelial cells to PBMC similar to the parental virus. However, subsequent flow chamber assay with infected PBMC showed reduced virus transfer to EC. It is very clear that gene deletions affected only the virus transfer step to EC, but not from the epithelium to the PBMC. The process of cell-to-cell virus transfer from infected epithelial cells to PBMC is relatively simple. EHV-1 undergoes full replication cycles in the epithelium and the released infectious virus particles result in dendritic cell infection [81,82,83]. Since the mutant viruses can replicate to levels comparable to parental viruses (Figure 2A–C), it was not surprising to see no effect on virus spread between epithelial cells and PBMC. On the other hand, virus replication in PBMC with subsequent egress is not fully identified. In addition, virus transfer between PBMC to EC is very complex and requires the interplay of several viral and cellular molecules [31,32,84]. We assume that these viral proteins (*ORF2* and *ORF17*) are involved in modulating several pathways in PBMC, including cell signaling, adhesion, and immune pathways, thereby *ORF2* and *ORF17* gene deletions resulted in reduced virus transfer to EC. Our findings clearly correlate with previous in vivo findings where *ORF2*, *ORF1*/*ORF71*, and *ORF1*/*ORF2* deletion mutants showed respiratory infection, comparable viremia, but no noticeable endothelial cell infection in four different experimental studies in horses [48,49,50,76].

It was shown before that all three PBMC subpopulations could be infected with EHV-1 [18,20,85,86]; however, identification of which subpopulation transfers the virus to EC is not clearly established. Our flow chamber assay showed that all three subpopulations of PBMC (T-, B-lymphocytes, and monocytes) could transfer the virus to EC. Gene deletions affected virus transfer from each PBMC subpopulation to levels comparable to the whole PBMC populations. We surmised that these viral proteins might target the same mechanisms in each subpopulation of PBMC.

EHV-1 undergoes limited virus replication in PBMC [32,85], but detailed knowledge regarding the expression of viral proteins and virus-induced changes in host pathways is limited [87]. Although several proteomic approaches have been reported for other herpesviruses, data regarding EHV-1-induced widespread changes in the host cell proteome is lacking [88,89]. In our proteomic analysis, we quantified the expression of 45 viral proteins in infected PBMC that includes structural and nonstructural proteins belonging to immediate-early, early and late expression kinetics. Expression of ORF2, but not ORF1 and ORF17 proteins was observed in parental EHV-1-infected PBMC. Our previous study revealed that in cell culture, expression of ORF17 and ORF1 was detectable from 2 and 4 h post-infection, but decreased after 8 and 16 h post-infection, respectively. Expression of ORF17 protein was not detected after 8 h post-infection [9,40]. This can be correlated with the absence of ORF17 and ORF1 in infected PBMC at 24 hpi. ORF2 protein was not detected in PBMC infected with Ab4∆ORF2 and Ab4∆ORF1/ORF2/ORF17 deletion mutant.

Parental EHV-1 infection upregulated proteins associated with several host pathways including MAPK, Ras signaling, endocytosis, oxidative phosphorylation, lysosomal pathways but downregulated herpesvirus and spliceosome pathways. MAPK pathway is involved in the manipulation of cellular functions such as signal transduction, cell adhesion, virus replication in target cells, and cell survival [90,91]. Ras-signaling pathway is implicated in sequential phosphorylation of at least 20 downstream molecules which transduce the signals from cell surface to nucleus including MAPK, c-Jun amino-terminal kinases (JNK) which is essential for cell survival from apoptosis [92]. Endocytosis pathway plays an essential role in regulating levels of many essential surface proteins such as G-protein coupled receptors, receptor tyrosine kinases and adhesion molecules, and transporters [93]. Herpesvirus infection pathway is mediated by numerous host and viral proteins which includes several pathways such as toll-like receptor signaling, pro-inflammatory cytokines, inhibition of apoptosis, antigen processing and presentation, JAK-STAT, calcium signaling, nuclear factor-kappa B (NF-κB), and B cell receptor signaling pathways [28,94,95]. Similar findings have been reported for other herpesviruses such as HSV, human cytomegalovirus virus, and Kaposi’s sarcoma-associated herpesvirus [92,96,97,98,99,100]. Upregulation of endocytosis and Ras signaling pathways and role of MAPK pathway in EHV-1 infection have been reported before [28,101,102,103].

While comparing the proteomic profile of Ab4-wt and mutants infected PBMC, *ORF1*, *ORF2*, and *ORF17* gene deletions have modulated several pathways, mainly chemokine signaling, MAPK, herpesvirus infection, and oxidative phosphorylation pathways. Ab4-wt infection downregulated herpesvirus infection pathway, while Ab4∆ORF1/ORF2/ORF17 mutant virus upregulated this pathway. Similar to the single gene deletion (Ab4∆ORF1 and Ab4∆ORF2), triple gene deletions modulated several pathways including chemokine signaling pathways. A previous in vitro study showed the potential role of MAPK pathway in EHV-1-infected PBMC. Inhibition of this pathway with a chemical inhibitor reduced virus transfer from infected monocytes to EC [28]. In our study, *ORF17* and *ORF2* gene deletions downregulated the proteins associated with MAPK pathway and showed reduced virus transfer to EC. We surmise that ORF17 and ORF2 viral proteins are involved in upregulating the MAPK pathway thereby facilitating virus transfer to EC. As described earlier, MAPK is essentially involved in transmitting various extracellular signals that induce cellular proliferation, differentiation, and survival [104]. It has been reported that herpesvirus infection alters MAPK signaling to promote virus internalization, dysregulate the cell cycle, regulate viral replication, and prevent host-cell death [105,106]. Studying the role of *ORF17* and *ORF2* in modulating MAPK pathway will be the scope of our future studies.

Parental EHV-1 infection greatly reduced cytokine and chemokine release. Higher levels of FGF-2 in Ab4-wt infected PBMC can be correlated with activation of MAPK pathway as confirmed by proteomic analysis. FGF-2 results in the activation of Ras-MAPK pathway, which is essential for virus spread [107,108]. Previous in vivo studies following experimental infection with Ab4-wt (neuropathogenic) strain showed release of fewer cytokines/chemokines at low concentration (IL-10, CCL2, CCL3) in comparison to RacL11 and NY03 strains (abortigenic strains) [109,110]. As expected from proteomic analysis, cytokine/chemokine estimation assay confirmed deletion of viral genes in altering cytokine/chemokine response. *ORF1* deletion showed release of more cytokines in infected PBMC. Similar findings were confirmed by mRNA expression, cytokine quantification, and migration assay [38]. Furthermore, IFNγ levels were higher in all *ORF1* deletion mutants. IFNγ is produced by antigen-activated leukocytes. Secreted IFNγ activates antigen-specific immunity, innate-cell mediated immunity, and cytokines/chemokines release. Ab4-wt infection blocks IFNγ release from PBMC and deleting *ORF1* gene resulted in release of more cytokines/chemokines which shows the immune-modulating potential of *ORF1* in Ab4 strain. *ORF2* gene deletion also showed release of more cytokines in both in vitro and in vivo [49,76]. *ORF2* gene deletion resulted in expression of higher levels of IL-1β cytokine, which is considered as a host defense against virus infection. It is a well-known immune evasion strategy employed by HSV-1 to retain IL-1β in the intracellular space of infected macrophages without being released to the extracellular space by blocking the function of caspase-1, which blocks the pro-inflammatory activity of IL-1β [111,112]. We presume that *ORF2* of EHV-1 also may have a similar function, thereby Ab4-wt infection in PBMC results in decreased release of IL-1β, while *ORF2* deletion restores expression. The role of *ORF2* in modulating of IL-1β secretion could be the potential area for further investigation. *ORF17* gene deletion did not restore cytokine/chemokine release completely, however, released more cytokines than parental EHV-1.

RacL11 strain, which is poorly spread to EC, naturally lacks *ORF1* and *ORF2* in its genome in comparison to Ab4-wt and showed release of more cytokines upon infecting PBMC [109]. Similarly, deletion of *ORF1* and *ORF2* genes in Ab4 restored expression of more cytokines, which clearly demonstrates that *ORF1* and *ORF2* have a major role in cytokine production.

## 5. Conclusions

In conclusion, we have identified and confirmed immune evading and cytokine modulating properties of different viral proteins of herpesviruses. Based on our flow chamber, proteomics, and cytokine/chemokine assay, all three targeted genes were multifunctional in nature. Nevertheless, these viral proteins are involved in more than one pathway in PBMC. We presume that *ORF1* probably plays a major role in Ras-signaling, chemokine signaling, and cell adhesion pathways. ORF2 and *ORF17* are implicated in chemokine signaling and MAPK signaling pathways thereby facilitating cell-to-cell virus spread. Furthermore, *ORF1*, *ORF2*, and *ORF17* deletion mutants (alone or in combinations) were capable of stimulating strong cytokine responses in vitro. These genes could be potential targets for the development of live attenuated vaccine therapeutics against EHV-1 infection in equines. In addition, identifying possible interactions and finding interaction partners for ORF1, ORF2, and ORF17 proteins including viral and cellular proteins are the potential area of future studies.

## Figures and Tables

**Table 1:** List of primers used for construction of mutant and revertant equine herpesvirus type 1 (EHV-1).

Primer	Primer Name	Nucleotide Sequence
**P1**	*ORF17* STOP Fwd	caaaggttggcttgctacatcaaggttatcaatcatgatgtaacagccagatagagagcccggtagggataacagggtaatcgat
**P2**	*ORF17* STOP Rev	gcaccagacacgagtcttcaccgggctctctatctggctgttacatcatgattgataaccttgccagtgttacaaccaattaacc
**P3**	*ORF17* pre For seq	ctttatgtgaattcaccgac
**P4**	*ORF17* post Rev seq	gttttatgactaatacctgg
**P5**	*ORF17* Revertant Fwd	caaaggttggcttgctacatcaaggttatcaatcatgatgtaccagccagatagagagcccggtagggataacagggtaatcgat
**P6**	*ORF17* Revertant Rev	gcaccagacacgagtcttcaccgggctctctatctggctggtacatcatgattgataaccttgccagtgttacaaccaattaacc
**P7**	*ORF1* deletion Fwd	tccacctgcaccttttccatctcctctccaactcgccgccaacgactgtagtaccgcaaaaggatgacgacgataagtaggg
**P8**	*ORF1* deletion Rev	aaaaataaatgcgattaacctttgcggtactacagtcgttggcggcgagttggagaggagcaaccaattaaccaattctgattag
**P9**	*ORF1* pre Fwd seq	ggctcctcccttttggctctgg
**P10**	*ORF1* post Rev seq	tctggtgctgatcggaatagtgta
**P11**	*ORF1* BamH Fwd	attggatccatgagacccgagggagtttc
**P12**	*ORF1* EcoRI Rev	cacgaattcttatttctccttcttgccgt
**P13**	*ORF1* kana Fwd	atttagccttccgctcctgtctgcttacactttacacttttctgctcgtcatgagacccgagggagtttc
**P14**	*ORF1* kana Rev	aggggtgtttgtgaaaataaacataatacaactgtgttgaaccacttgttttatttctccttcttgccgt
**P15**	*ORF1* Revertant Fwd	ttccactttctccacctgcaccttttccatctcctctccaactcgccgccatgagacccgagggagtttc
**P16**	*ORF1* Revertant Rev	gagtgcatgtaaaaataaatgcgattaacctttgcggtactacagtcgttttatttctccttcttgccgt
**P17**	*ORF2* deletion For	aaaacgactgtagtaccgcaaaggttaatcgcatttatttgcttaaacactttggagcgaaggatgacgacgataagtaggg
**P18**	*ORF2* deletion Rev	cgcccccataccccgccccctcgctccaaagtgtttaagcaaataaatgcgattaaccttcaaccaattaaccaattctgattag
**P19**	*ORF2* pre Fwd seq	taacaaacggcaagaaggag
**P20**	*ORF2* post Rev seq	taacgctgtagattgagttt
**P21**	*ORF2* EcoRI Fwd	aattagaattcttacatgcactcctttccaa
**P22**	*ORF2* Xba Rev	atatatctagaatggatccagcgtggaggag
**P23**	*ORF2* Kan Fwd	cgcggggcggccgcactaccatcggaagtttaccaggatgacgacgataagtaggg
**P24**	*ORF2* Kan Rev	ggtagtgcggccgccccgcggtgatttctagtaacaaccaattaaccaattctgattag
**P25**	*ORF2* Revertant Fwd	aaggagaaataaaacgactgtagtaccgcaaaggttaatcgcatttatttttacatgcactcctttccaa
**P26**	*ORF2* Revertant Rev	ttcaggcatacgcccccataccccgccccctcgctccaaagtgtttaagcatggatccagcgtggaggag

**Table 2:** Ab4-wt infection of PBMC subpopulations. The data represents the mean ± SD of three independent and blinded experiments.

Cell Marker	Cell	% in PBMC	Rate of Infection in %	% in Infected Population
**CD14**	Monocyte	27.1 ± 1.7	41.3 ± 2.3	66.2 ± 1.1
**IgM**	B lymphocyte	9.8 ± 1.1	22 ± 3.2	12.9 ± 0.8
**CD3**	T lymphocyte	63.2 ± 2.3	5.5 ± 0.5	20.8 ± 1.5

**Table 3:** Classification of EHV-1 proteins quantified in infected PBMC at 24 hpi.

Type	Name of Viral Proteins	
**Nonstructural protein**	Ribonucleoside-diphosphate reductase R1 Ribonucleoside-diphosphate reductase R2 DNA polymerase Uracil-DNA glycosylase Alkaline nuclease Major viral transcription factor Serine/theonine-protein kinase Major DNA-binding protein mRNA export factor ICP27	Nuclear egress protein 1 Nuclear egress protein 2 DNA polymerase processivity factor DNA primase Thymidine kinase Packaging protein UL32 Tripartite terminase Deoxyuridine 5-triphosphate Internal repeat 6
**Structural protein**	**Tegument proteins** Tegument protein UL47 Tegument protein UL46 Tegument protein VP16 Large tegument protein Inner tegument protein Tegument protein VP22 Tegument protein UL21	Serine/theonine-protein kinase UL13 Cytoplasmic envelopment protein 2 Envelope protein UL45 E3 ubiquitin-protein ligase ICP0
	**Capsid proteins** Major capsid protein Triplex capsid protein 1 Triplex capsid protein 2	Capsid vertex component Capsid vertex component 1 Portal protein
	**Envelope proteins** Glycoprotein G Glycoprotein I Glycoprotein D Glycoprotein H	Glycoprotein B Glycoprotein C Glycoprotein E
**Uncharacterized proteins**	ORF protein 2 ORF protein 59 ORF protein 3	

**Table 4:** Pathways differentially regulated in Ab4-wt- and mutant viruses-infected PBMC. PBMC were infected with Ab4-wt or mutant viruses at an MOI of 1. At 24 hpi, infected PBMC were sorted and proteomic analysis was performed. Pathways significantly upregulated and downregulated (based on *p*-value and Benjamini-corrected Fisher’s exact test) in infected PBMC in comparison to non-infected PBMC are given; (*n* = 4).

Infected Population	Pathways Upregulated	Pathways Downregulated
**PBMC** **vs.** **Ab4-wt**	Lysosome cAMP signaling Ras signaling pathway Endocytosis Platelet activation Leukocyte transendothelial migration Oxydative phosphorylation Fatty acid elongation	Herpesvirus infection Spliceosome Chemokine signaling pathway RNA degradation Apoptosis
**PBMC** **vs.** **Ab4∆ORF17**	Lysosome Herpesvirus infection Oxydative phosphorylation Protein processing in endoplasmic reticulum Fc epsilon RI signaling Metabolic pathways	Chemokine signaling pathway MAPK signaling pathway Spliceosome RNA transport
**PBMC** **vs.** **Ab4∆ORF1**	mTOR signaling pathway Endocytosis Lysosome Focal adhesion Ras signaling pathway Herpesvirus infection Chemokine signaling pathway Leukocyte transendothelial migration Platelet activation	Spliceosome RNA degradation Metabolic pathways Aminoacyl-tRNA biosynthesis
**PBMC** **vs.** **Ab4∆ORF 2**	Herpesvirus infection mTOR signaling pathway Regulation of actin cytoskeleton Chemokine signaling pathway	Spliceosome RNA degradation MAPK signaling pathway
**PBMC** **vs.** **Ab4∆ORF 1/ORF2/ORF17**	Endocytosis Lysosome Herpesvirus infection T cell signaling Chemokine signaling pathway	Spliceosome RNA degradation MAPK signaling pathway

**Table 5:** Compiled cytokine and chemokines profile.

PBMC Samples	Number of Detected Cytokines			Co-Culture Samples	Number of Detected Cytokines	
	3 hpi	6 hpi	12 hpi		3 hpi	6 hpi
PBMC	5	5	9	PBMC-EC	8	10
Ab4-wt	3	1	4	Ab4-wt-EC	5	6
Ab4∆ORF17	3	4	4			
Ab4∆ORF1	3	4	7			
Ab4∆ORF2	4	4	8			
Ab4∆ORF17/ORF2	4	6	4			
Ab4∆ORF1/ORF2	3	5	7			
Ab4∆ORF1/ORF17	3	4	7			
Ab4∆ORF1/ORF2/ORF17	3	4	7			

## Supplementary Materials

The following are available online at https://www.mdpi.com/1999-4915/12/9/999/s1, Figure S1: Rate of infection of PBMC with Ab4-wt and mutant viruses; Table S1: Complete list of proteins differentially regulated in corresponding pathways in Ab4-wt and mutant viruses infected PBMC; Table S2: Function and subcellular localization Ab4-viral proteins quantified in infected PBMC; Table S3: LFQ intensity of each viral proteins detected in PBMC infected with Ab4-wt and mutant EHV-1 at 24 hpi; Table S4: Overview of cytokine produced, their cell source, targets, and functions.
