# Supplementary material for: Equine Herpesvirus Type 1 Modulates Cytokine and Chemokine Profiles of Mononuclear Cells for Efficient Dissemination to Target Organs
Source: Viruses. 2020 Sep 8;12(9):999. doi: 10.3390/v12090999 (PMC7551999; doi:10.3390/v12090999)
Supplement: Supplementary file 1 [file viruses-12-00999-s001.pdf]

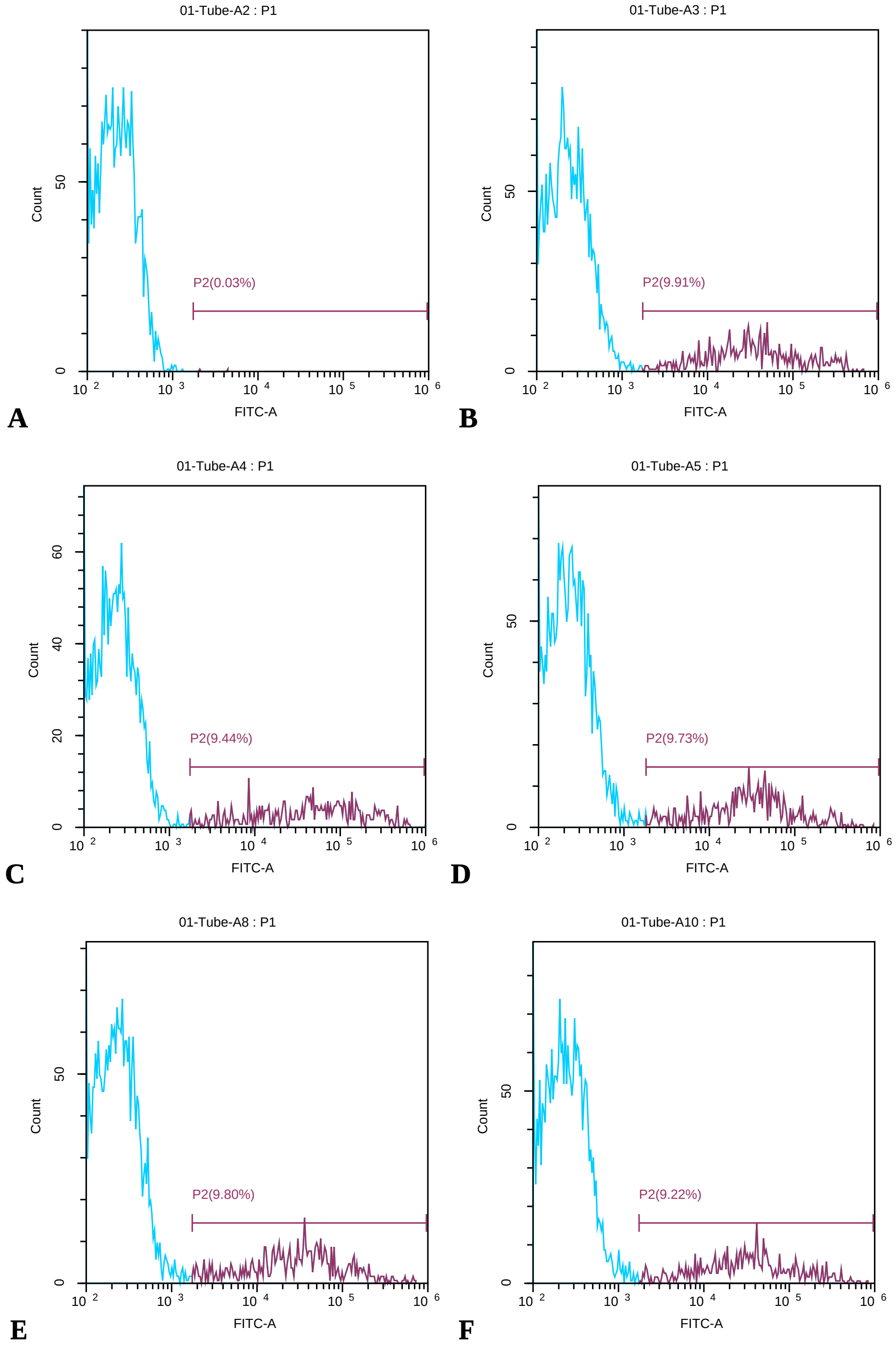

Supplementary figure S1: Rate of infection of PBMC with Ab4-wt and mutant viruses. PBMC were infected with Ab4-wt or mutant viruses at an MOI of 0.1 for 24 h. Infected PBMC were sorted based on *egfp* expression using FACSAria (BD Bioscience) and used for transwell co-cultivation and flow chamber experiments. (A) mock infected (B) Ab4-wt, (C) Ab4ΔORF1 (D) Ab4ΔORF2 (E) Ab4ΔORF17) and (F) Ab4Δ/ORF1/ORF2/ORF17 infected PBMC at 24 hrs post infection. P2 indicates percentage of *egfp* positive PBMC. (n=4)

Infected population	Pathways upregulated		Pathways downregulated	
	**Pathways**	**Genes**	**Pathways**	**Genes**
**PBMC Vs Ab4-wt**	**Lysosome**	ATPase H+ transporting V0 subunit d1<br>Adaptor related protein complex 3 mu 1 subunit<br>Adaptor related protein complex 3 mu 2 subunit<br>Scavenger receptor class B member 2	**Herpesvirus infection**	CD58 molecule<br>Proteasome 26S subunit, non-ATPase 2<br>recombination signal binding protein for immunoglobulin kappa J region like<br>recombination signal binding protein for immunoglobulin kappa J region
	**Ras signaling pathway**	RAP1A, member of RAS oncogene family<br>Phosphoinositide-3-kinase regulatory subunit 1<br>Ras-related protein Rap-1A		
	**Endocytosis**	EH domain containing 1<br>EH domain containing 3<br>RAB35, member RAS oncogene family<br>RAB5A, member RAS oncogene family	**Spliceosome**	LSM6 homolog, U6 small nuclear RNA and mRNA degradation associated<br>RNA binding motif protein 22<br>Cell division cycle 40<br>Squamous cell carcinoma antigen recognized by T-cells 1
	**Platelet activation**<br>**Leukocyte transendothelial migration**	RAP1A, member of RAS oncogene family<br>Phosphoinositide-3-kinase regulatory subunit 1<br>Ras-related protein Rap-1A	**Chemokine signaling pathway**	RAP1A, member of RAS oncogene family<br>Mitogen-activated protein kinase kinase 1<br>Stress induced phosphoprotein 1
	**Oxidative phosphorylation**	ATP synthase, H+ transporting, mitochondrial F1 complex, alpha subunit 1<br>ATPase H+ transporting V0 subunit d1<br>NADH:ubiquinone oxidoreductase core subunit V1	**RNA degradation**	TNFRSF1A associated via death domain<br>Chromodomain helicase DNA binding protein 4<br>LSM6 homolog, U6 small nuclear RNA and mRNA degradation associated<br>RNA binding motif protein 22
	**Fatty acid elongation**	Hydroxysteroid 17-beta dehydrogenase 12<br>Mitochondrial trans-2-enoyl-CoA reductase	**Apoptosis**	Apoptosis inducing factor, mitochondria associated 1<br>Caspase 1, apoptosis-related cysteine peptidase<br>Caspase 10<br>MALT1 paracaspase
	**Herpesvirus infection**	CD58 molecule<br>TNFRSF1A associated via death domain<br>Proteasome 26S subunit, non-ATPase 12	**Chemokine signaling pathway**	RAP1A, member of RAS oncogene family<br>Mitogen-activated protein kinase kinase 1<br>Ras-related protein Rap-1A

		Recombination signal binding protein for immunoglobulin kappa J region like<br>Recombination signal binding protein for immunoglobulin kappa J region l		Stress induced phosphoprotein 1
**PBMC Vs Ab4ΔORF17**	**Lysosome**	ATPase H+ transporting V0 subunit d1<br>Adaptor related protein complex 3 mu 1 subunit<br>Adaptor related protein complex 3 mu 2 subunit<br>Scavenger receptor class B member 2	**MAPK signaling pathway**	Protein phosphatase 5 catalytic subunit<br>RAP1A, member of RAS oncogene family<br>Mitogen-activated protein kinase kinase 1<br>Ras-related protein Rap-1A
	**Oxidative phosphorylation**	ATP synthase, H+ transporting, mitochondrial F1 complex, alpha subunit 1<br>ATPase H+ transporting V0 subunit d1<br>NADH:ubiquinone oxidoreductase core subunit V1	**RNA transport**	RNA binding motif protein 22<br>Pre-mRNA processing factor 3<br>NOP2/Sun RNA methyltransferase family member 2<br>Ribosomal RNA processing 1B
	**Protein processing in endoplasmic reticulum and Fc epsilon RI signaling**	ER membrane protein complex subunit 1<br>Endoplasmic reticulum lectin 1<br>Serine/threonine-protein phosphatise 6 catalytic subunit<br>Receptor-type tyrosine-protein phosphatase epsilon	**Spliceosome**	LSM6 homolog, U6 small nuclear RNA and mRNA degradation associated<br>RNA binding motif protein 22<br>Cell division cycle 40<br>Squamous cell carcinoma antigen recognized by T-cells 1
	**Metabolic pathways**	24-dehydrocholesterol reductase<br>T-cell immune regulator 1, ATPase H+ transporting V0 subunit a3<br>Acetyl-CoA acetyltransferase 2<br>Acyl-CoA synthetase long-chain family member 3<br>Adenosylhomocysteinase like 1<br>Adenosylhomocysteinase like 2<br>Branched chain amino acid transaminase 2<br>Ceramide synthase 2<br>Inositol polyphosphate-1-phosphatase<br>Methylcrotonoyl-CoA carboxylase 1<br>Methylcrotonoyl-CoA carboxylase 2<br>Phosphatidylinositol glycan anchor biosynthesis class U<br>Phospholipase B1		

		Pyruvate dehydrogenase complex component		
**PBMC Vs Ab4ΔORF1**	**mTOR signaling pathway**	3-phosphoinositide dependent protein kinase 1<br>Ras related GTP binding C<br>Ras related GTP binding D<br>Phosphatidylinositol-4,5-bisphosphate 3-kinase catalytic subunit gamma	**Metabolic pathways**	ATP synthase, H+ transporting, mitochondrial F1 complex, epsilon subunit (ATP5E)<br>ATPase H+ transporting V0 subunit a1<br>NADH:ubiquinone oxidoreductase core subunit V1<br>Aconitase 1<br>Fucokinase<br>Galactosidase beta 1<br>Hydroxysteroid 17-beta dehydrogenase 12<br>Mannosidase alpha class 2A member 1<br>Thymidine phosphorylase
	**Herpesvirus infection**	CD58 molecule<br>Phosphatidylinositol-4,5-bisphosphate 3-kinase catalytic subunit gamma<br>Proteasome 26S subunit, non-ATPase 12		
	**Lysosome**	ATPase H+ transporting V0 subunit d1<br>Adaptor related protein complex 3 mu 1 subunit<br>Adaptor related protein complex 3 mu 2 subunit<br>Scavenger receptor class B member 2		
	**Chemokine signaling pathway**	RAP1A, member of RAS oncogene family<br>Mitogen-activated protein kinase kinase 1<br>Ras-related protein Rap-1A<br>Stress induced phosphoprotein 1	**Spliceosome**	LSM6 homolog, U6 small nuclear RNA and mRNA degradation associated<br>RNA binding motif protein 22<br>Cell division cycle 40<br>Squamous cell carcinoma antigen recognized by T-cells 1
	**Platelet activation Leukocyte transendothelial migration**	RAP1A, member of RAS oncogene family<br>Phosphoinositide-3-kinase regulatory subunit 1<br>Ras-related protein Rap-1A<br>CD58 molecule	**RNA degradation**	TNFRSF1A associated via death domain<br>Chromodomain helicase DNA binding protein 4<br>LSM6 homolog, U6 small nuclear RNA and mRNA degradation associated<br>RNA binding motif protein 22
	**Focal adhesion and Ras signaling pathway**	RAP1A, member of RAS oncogene family<br>Phosphoinositide-3-kinase regulatory subunit 1<br>Ras-related protein Rap-1A	**Aminoacyl-tRNA biosynthesis**	Histidyl-tRNA synthetase 2, mitochondrial<br>Histidyl-tRNA synthetase<br>phenylalanyl-tRNA synthetase beta subunit
	**Endocytosis**	EH domain containing 1<br>EH domain containing 3<br>RAB35, member RAS oncogene family		

**PBMC Vs Ab4ΔORF2**	**Herpesvirus infection**	CD58 molecule<br>proteasome 26S subunit, non-ATPase 12<br>proteasome 26S subunit, non-ATPase 2	**Spliceosome**	LSM6 homolog, U6 small nuclear RNA and mRNA degradation associated<br>RNA binding motif protein 22<br>Cell division cycle 40<br>Squamous cell carcinoma antigen recognized by T-cells 1
	**mTOR signaling**	3-phosphoinositide dependent protein kinase 1<br>Ras related GTP binding C<br>Ras related GTP binding D<br>Phosphatidylinositol-4,5-bisphosphate 3-kinase catalytic subunit gamma		
	**Regulation of actin cytoskeleton**	FYVE, RhoGEF and PH domain containing 3<br>Integrin subunit beta<br>Phosphatidylinositol-4,5-bisphosphate 3-kinase catalytic subunit gamma<br>Phosphatidylinositol-5-phosphate 4-kinase type 2 gamma<br>Protein phosphatase 1 regulatory subunit 12A	**RNA degradation**	TNFRSF1A associated via death domain<br>Chromodomain helicase DNA binding protein 4<br>LSM6 homolog, U6 small nuclear RNA and mRNA degradation associated<br>RNA binding motif protein 22
	**Chemokine signaling pathway**	RAP1A, member of RAS oncogene family<br>Mitogen-activated protein kinase kinase 1<br>Ras-related protein Rap-1A<br>Stress induced phosphoprotein 1	**MAPK signaling pathway**	RAP1A, member of RAS oncogene family<br>Interleukin 1 alpha<br>Protein phosphatase 5 catalytic subunit<br>Ras-related protein Rap-1A<br>Ribosomal protein S6 kinase A5
**PBMC Vs**	**Endocytosis**	EH domain containing 1<br>EH domain containing 3<br>RAB35, member RAS oncogene family<br>RAB5A, member RAS oncogene family	**Spliceosome**	LSM6 homolog, U6 small nuclear RNA and mRNA degradation associated<br>RNA binding motif protein 22<br>Cell division cycle 40<br>Squamous cell carcinoma antigen recognized by T-cells 1
	**Herpesvirus infection**	CD58 molecule<br>proteasome 26S subunit, non-ATPase 12<br>proteasome 26S subunit, non-ATPase 2		
	**Lysosome**	ATPase H+ transporting V0 subunit d1<br>Adaptor related protein complex 3 mu 1 subunit<br>Adaptor related protein complex 3 mu 2 subunit<br>Scavenger receptor class B member 2	**MAPK signaling pathway**	RAP1A, member of RAS oncogene family<br>Interleukin 1 alpha<br>Protein phosphatase 5 catalytic subunit<br>Ras-related protein Rap-1A<br>Ribosomal protein S6 kinase A5

**Ab4ΔORF1/ ORF2/ORF17**	**Chemokine signaling pathway**	RAP1A, member of RAS oncogene family<br>Mitogen-activated protein kinase kinase 1<br>Ras-related protein Rap-1A<br>Stress induced phosphoprotein 1	**RNA degradation**	TNFRSF1A associated via death domain<br>Chromodomain helicase DNA binding protein 4<br>LSM6 homolog, U6 small nuclear RNA and mRNA degradation associated<br>RNA binding motif protein 22
	**T cell signaling**	T-cell immune regulator 1, ATPase H+ transporting V0 subunit a3<br>Squamous cell carcinoma antigen recognized by T-cells 1<br>Phosphatidylinositol-4,5-bisphosphate 3-kinase catalytic subunit gamma<br>Phosphatidylinositol-5-phosphate 4-kinase type 2 gamma		

Supplementary Table S1: Complete list of proteins differentially regulated in corresponding pathways in Ab4-wt and mutant viruses infected PBMC. PBMC were infected with Ab4-wt and mutants at MOI of 1. At 24 hpi, infected PBMC were sorted and proteomic analysis was performed. Pathways significantly upregulated and downregulated (based on *p*-value and Benjamini-corrected modified Fisher's exact test value) in infected PBMC in comparison healthy PBMC were given (n=4).

S. No	Uniprot ID	Protein names	Gene/ ORF	Function	Subcellular location
**1**	P28846	Ribonucleoside-diphosphate reductase large subunit (R1)	RIR1/ ORF21	Precursors necessary for viral DNA synthesis. Allows virus growth in non-dividing cells	Host membrane
**2**	P28847	Ribonucleoside-diphosphate reductase small subunit (R2)	RIR2/ ORF20	Precursors necessary for viral DNA synthesis. Allows virus growth in non-dividing cells	Host membrane
**3**	P28858	DNA polymerase catalytic subunit	ORF30	Replicates viral genomic DNA	Host nucleus, replication compartments where viral DNA replication occurs.
**4**	P28866	Uracil-DNA glycosylase (UDG)	ORF61	Excises uracil residues from the DNA. Reduce deleterious uracil incorporation into the viral genome, in terminally differentiated cells which lack DNA repair enzymes.	Host nucleus
**5**	P28892	Deoxyuridine 5'-triphosphate nucleotidohydrolase (dUTPase)	DUT / ORF9	Nucleotide metabolism: decreases the intracellular concentration of dUTP to avoid uracil incorporation into viral DANN	
**6**	P28919	Alkaline nuclease	ORF50	Role in processing non linear or branched viral DNA intermediates to promote the production of mature packaged unit-length linear progeny viral DNA molecules. Exhibits endonuclease and exonuclease activities	Host nucleus, cytoplasm
**7**	P28920	Major capsid protein	ORF42	Self-assembles to form an icosahedral capsid with a T=16 symmetry	Virion, Host nucleus
**8**	P28921	Triplex capsid protein 2	TRX2/ ORF43	Structural component of the T=16 icosahedral capsid.	Virion, Host nucleus
**9**	P28925	Major viral transcription factor	ORF64	This IE protein is a multifunctional protein capable of migrating to the nucleus, binding to DNA, trans-activating other viral genes, and autoregulating its own synthesis.	Host nucleus
**10**	P28926	Serine/threonine-protein kinase US3 homolog	ORF69	Multifunctional serine/threonine kinase, role in egress of virus particles from the nucleus, modulation of the actin cytoskeleton and inhibition of apoptosis.	Host cytoplasm, Host nucleus
**11**	P28928	Capsid vertex component 2	CVC2/ ORF36	Capsid vertex-specific component that plays a role during viral DNA encapsidation, assuring correct genome cleavage and presumably stabilizing capsids that contain full-length viral genomes.	Virion, Host nucleus
**12**	P28929	Tegument protein UL47 homolog (GP10)	ORF13	Tegument protein that can bind to various RNA transcripts. Role in the attenuation of selective viral and cellular mRNA degradation by modulating the activity of host shutoff RNase UL41/VHS. Role in the primary envelopement of virions in the perinuclear space, by interacting with two nuclear egress proteins UL31 and UL34.	Virion tegument, Host nucleus, cytoplasm

**13**	P28932	Major DNA-binding protein (DBP)	ORF31	Crucial roles in viral infection. Participates in the opening of the viral DNA origin to initiate replication by interacting with the origin-binding protein	Host nucleus
**14**	P28935	Triplex capsid protein 1	TRX1/ ORF22	Structural component of the T=16 icosahedral capsid	Virion, Host nucleus
**15**	P28937	Tegument protein UL46 homolog	ORF14	Modulates alpha trans-inducing factor-dependent activation of alpha genes	Virion tegument, Host cell membrane
**16**	P28938	Tegument protein VP16 homolog (Alpha trans-inducing protein)	ORF12	Transcriptional activator of immediate-early (IE) gene products. Key activator of lytic infection by initiating the lytic program through the assembly of the transcriptional regulatory VP16-induced complex composed of VP16 and two cellular factors, HCFC1 and POU2F1. VP16-induced complex represents a regulatory switch: when it is on, it promotes IE-gene expression and thus lytic infection, and when it is off, it limits IE-gene transcription favoring latent infection.	Virion tegument, Host nucleus
**17**	P28939	mRNA export factor ICP27 homolog (Transcriptional regulator IE63 homolog)	ORF5	Multifunctional regulator of the expression of viral genes that mediates nuclear export of viral intronless mRNAs. This immediate early (EI) protein promotes the nuclear export of viral intronless mRNAs by interacting with mRNAs and host NXF1/TAP	Host cytoplasm, nucleus
**18**	P28944	Portal protein	ORF56	Forms a portal in the viral capsid through which viral DNA is translocated during DNA packaging. Assembles as a dodecamer at a single fivefold axe of the T=16 icosahedric capsid. Binds to the molecular motor that translocates the viral DNA, termed terminase.	Virion, Host nucleus
**19**	P28950	Capsid vertex component 1	CVC1/ ORF45	Role during viral DNA encapsidation, assuring correct genome cleavage and presumably stabilizing capsids that contain full-length viral genomes	Virion, Host nucleus
**20**	P28951	Nuclear egress protein 1	NEC1/ ORF29	Essential role in virion nuclear egress, the first step of virion release from infected cell	Host nucleus inner membrane through interaction with NEC2
**21**	P28954	Nuclear egress protein 2	NEC2/ ORF26	Essential role in virion nuclear egress, the first step of virion release from infected cell	Host nucleus inner membrane
**22**	P28955	Large tegument protein deneddylase	ORF24	Large tegument protein that plays multiple roles in the viral cycle	Virion tegument, cytoplasm, nucleus. Associated with capsid
**23**	P28956	Inner tegument protein	ORF23	Essential role in cytoplasmic secondary envelopment during viral egress.	Virion tegument, cytoplasm, nucleus, golgi apparatus
**24**	P28958	DNA-binding Polymerase accessory protein	ORF18	Essential role in viral DNA replication by acting as the polymerase accessory subunit	Host nucleus

**25**	P28960	Tegument protein VP22	ORF11	Tegument protein that plays different roles during the time course of infection. Participates in both the accumulation of viral mRNAs and viral protein translation at late time of infection. Most abundant tegument protein (2000 copies per virion).	Virion tegument, Host cytoplasm, nucleus, Golgi apparatus
**26**	P28962	DNA primase	ORF7	Essential component of the helicase/primase complex. Unwinds the DNA at the replication forks and generates single-stranded DNA for both leading and lagging strand synthesis	Host nucleus
**27**	P28966	Serine/threonine-protein kinase UL13 homolog	ORF49	Multifunctional serine/threonine kinase, role in egress of virus particles from the nucleus, modulation of the actin cytoskeleton and regulation of viral and cellular gene expression. Regulates the nuclear localization of viral envelopment factors UL34 and UL31 homologs, by phosphorylating the US3 kinase homolog, indicating a role in nuclear egress. Disrupts host nuclear lamins, including LMNA and LMNB1. Phosphorylates the viral Fc receptor composed of glycoproteins E (gE) and I (gI). Phosphorylation of glycoprotein E (gE) by UL13 homolog alters its subcellular localization, from the host early endosome to the plasma membrane.	Virion tegument, Host nucleus
**28**	P28967	Envelope glycoprotein G (gG)	gG/ ORF70	Chemokine-binding protein that inhibits neutrophils' chemotaxis.	Virion membrane, Single-pass membrane protein
**29**	P28970	Cytoplasmic envelopment protein 2	ORF46	Plays a critical role in cytoplasmic virus egress. Participates in the final step of tegumentation and envelope acquisition within the host cytoplasm by directly interacting with the capsid. Upon virion binding to target cell, a signaling cascade is triggered to disrupt the interaction with the capsid, thereby preparing capsid uncoating.	Virion tegument, Host cytoplasm, nucleus
**30**	P28972	Tegument protein UL21 homolog	ORF40	May participate in DNA packaging/capsid maturation events. Promotes efficient incorporation of tegument proteins UL46, UL49, and US3 homologs into virions. May also play a role in capsid transport to the trans-Golgi network (TGN) (By similarity).	Virion tegument, Host cytoplasm, nucleus
**31**	P28979	Gene 2 protein	ORF2	Immunomodulatory protein	
**32**	P28981	Envelope protein UL45 homolog	ORF15	Uncharacterized	Virion membrane, Single-pass type II membrane protein
**33**	P28983	Uncharacterized gene 59 protein	ORF59	Uncharacterized	
**34**	P28988	Gene 3 protein	ORF3	Uncharacterized	
**35**	P28990	E3 ubiquitin-protein ligase ICP0	ORF63	Evades nuclear antiviral defenses triggered by dsDNA viruses. Acts during the initial stages of lytic infection and the reactivation of latent viral genome. Prevents the antiviral effect of nuclear bodies by degrading host PML and SP100	
**36**	P69185	Thymidine kinase	TK/ ORF38	Catalyzes the transfer of the gamma-phospho group of ATP to thymidine to generate dTMP in the salvage pathway of pyrimidine synthesis. The dTMP - substrate for DNA polymerase in viral DNA replication. Allows virus to be reactivated and to grow in non-proliferative cells lacking a high concentration of phosphorylated nucleic acid precursors.	
**37**	P69329	Packaging protein UL32 homolog	ORF28	Role in efficient localization of neo-synthesized capsids to nuclear replication compartments, thereby controlling cleavage and packaging of virus genomic DNA.	Host cytoplasm, nucleus

**38**	Q6DLD8	Envelope glycoprotein I (gI)	gI/ ORF73	In epithelial cells, the heterodimer gE/gI is required for the cell-to-cell spread of the virus, by sorting nascent virions to cell junctions	Virion membrane, Host cell membrane, cell junction, Golgi apparatus membrane
**39**	Q6DLD9	Envelope glycoprotein D (gD) (Glycoprotein 17/18)	gD/ ORF72	Envelope glycoprotein that binds to host cell entry receptors. May trigger fusion with host membrane, by recruiting the fusion machinery composed of gB and gH/gL	Virion membrane, endosomes and trans-Golgi
**40**	Q6DLH1	Envelope glycoprotein H (gH)	gH/ ORF39	The heterodimer glycoprotein H-glycoprotein L is required for the fusion of viral and plasma membranes leading to virus entry into the host cell.	Virion membrane, Host cell membrane, endosome membrane
**41**	Q6DLH8	Envelope glycoprotein B (gB)	gB/ ORF33	Envelope glycoprotein that forms spikes at the surface of virion envelope	Virion membrane, Host cell membrane, endosome membrane, Golgi
**42**	Q6DLH9	Tripartite terminase subunit 1	TRM1/ ORF32	Component of the molecular motor that translocates viral genomic DNA in empty capsid during DNA packaging. Forms a tripartite terminase complex with TRM2 and TRM3 in the cytoplasm. The complex reaches the host nucleus and interacts with capsid portal vertex. This portal forms a ring in which genomic DNA is translocated into the capsid. Role for the cleavage of concatemeric viral DNA into unit length genomes.	Host nucleus. Seen external surface of the viral capsid during assembly and DNA packaging, but absent in extracellular mature virions.
**43**	Q6LAQ8	Uncharacterized gene 67 protein (IR6 protein)	IR6/ ORF67	Determinant of EHV-1 virulence and play role in virus maturation and/or egress	Host cytoplasm
**44**	Q6S6Q5	Envelope glycoprotein C (gC) (Glycoprotein 13)	gC GP13/ ORF16	Essential for the initial attachment to heparan sulfate moities of the host cell surface proteoglycans. Plays also a role in host immune evasion by inhibiting the host complement cascade activation	Virion membrane
**46**	Q6S6V7	Envelope glycoprotein E (gE)	gE ORF/74	In epithelial cells, the heterodimer gE/gI is required for the cell-to-cell spread of the virus, by sorting nascent virions to cell junctions	Virion membrane, Host cell membrane, cell junction, Golgi, endosome membrane

Supplementary Table S2: Function and subcellular localization Ab4-viral proteins quantified in infected PBMC. PBMC were infected with Ab4-wt and mutant viruses, and proteomic analysis was performed at 24 hpi. (n=4). Data regarding function of viral proteins were from uniprot database.

S. No:	Viral protein Name	LFQ intensity					
		ORF	EHV-1 WT	EHV-1 ΔUL43	EHV-1 ΔORF1	EHV-1 ΔORF2	EHV-1 ΔUL43/ORF1/2
**1**	Ribonucleoside-diphosphate reductase	21	38457000	42760000	28937000	34619000	31547000
**2**	Ribonucleoside-diphosphate reductase	20	5598100	5476500	5585500	5649700	6021400
**3**	DNA polymerase	30	2382500	3150200	3580700	3538800	3046200
**4**	Uracil-DNA glycosylase	61	5891500	5534200	8313800	7703800	6132800
**5**	Deoxyuridine 5-triphosphate	9	2214900	1760400	2349300	2203100	2709400
**6**	Alkaline nuclease	50	13880000	15983000	13770000	15895000	15346000
**7**	Major capsid protein	42	5.59E+08	7.13E+08	4.05E+08	3.15E+08	4.34E+08
**8**	Triplex capsid protein 2	43	62978000	95072000	60136000	87828000	64373000
**9**	Major viral transcription factor	64	3.25E+08	82903000	6.86E+08	5.72E+08	82162000
**10**	Serine/threonine-protein kinase	69	30570000	40562000	42719000	37277000	32926000
**11**	Capsid vertex component	36	9458300	10320000	5259200	12150000	8031600
**12**	Tegument protein UL47	13	59701000	95905000	48184000	74667000	82758000
**13**	Major DNA-binding protein	31	4.15E+08	4.22E+08	4.44E+08	3.27E+08	4.39E+08
**14**	Triplex capsid protein 1	22	76925000	80867000	49053000	85817000	74079000
**15**	Tegument protein UL46	14	8763400	11152000	8561200	12463000	9024200
**16**	Tegument protein VP16	12	25461000	29828000	24032000	32303000	33741000
**17**	mRNA export factor ICP27	5	65052000	45830000	48386000	41274000	62247000
**18**	Portal protein	56	2174600	3901800	3018400	3896100	3299100
**19**	Capsid vertex component 1	45	10922000	15555000	9218600	13059000	11478000
**20**	Nuclear egress protein 1	29	21609000	15946000	11739000	12550000	16998000
**21**	Nuclear egress protein 2	26	60217000	96829000	64970000	82555000	70709000
**22**	Large tegument protein	24	30209000	38959000	22967000	26724000	31338000
**23**	Inner tegument protein	23	27162000	31615000	23699000	30601000	26773000
**24**	DNA polymerase processivity factor	18	1.32E+08	1.45E+08	1.33E+08	1.53E+08	1.22E+08
**25**	Tegument protein VP22	11	3E+08	1.05E+08	72235000	1.11E+08	77521000
**26**	DNA primase	7	0	403560	0	416120	445360

**27**	Serine/threonine-protein kinase UL13	49	1029400	1267500	0	992370	1066300
**28**	Glycoprotein G	70	7763200	10651000	6170100	5697000	5825100
**29**	Cytoplasmic envelopment protein 2	46	5710100	6904300	5807900	5667900	5820700
**30**	Tegument protein UL21	40	19523000	13215000	10426000	10602000	40850000
**31**	ORF protein 2	2	6509700	13405000	2613700	0	0
**32**	Envelope protein UL45	15	3423900	6110500	2451900	4253500	4048600
**33**	ORF protein 59	59	12931000	10356000	10649000	10223000	10612000
**34**	ORF protein 3	3	5969100	8898100	7535900	3729600	0
**35**	E3 ubiquitin-protein ligase ICP0	63	10721000	11200000	9420800	12522000	15578000
**36**	Thymidine kinase	38	56063000	72649000	44132000	67118000	64495000
**37**	Packaging protein UL32	28	0	0	799790	0	1261800
**38**	Glycoprotein I	73	13318000	14348000	10520000	12638000	13165000
**39**	Glycoprotein D	72	11519000	17086000	0	9276600	9266900
**40**	Glycoprotein H	39	11487000	24548000	10290000	16199000	12927000
**41**	Glycoprotein B	33	1.2E+08	2.15E+08	74321000	1.46E+08	1.4E+08
**42**	Tripartite terminase	32	747020	797000	725740	813340	755250
**43**	Internal repeat 6	67	3.06E+08	6.6E+08	4.3E+08	5.31E+08	2.38E+08
**44**	Glycoprotein C	16	34061000	82703000	20409000	58136000	46296000
**45**	Glycoprotein E	74	7132200	13009000	7924400	8334700	8958100

Supplementary Table S3: LFQ intensity of each viral proteins detected in PBMC infected with Ab4-wt and mutant EHV-1 at 24 hpi.

Cytokine	Cell source	Targets	Functions	Reference
**G-CSF**	Monocytes, endothelial cells, fibroblasts	Stem cells in bone marrow	Granulocyte production	[1,2]
**IL-1α and IL-1β**	Macrophages, B cells and dendritic cells	T and B cells, NK cells	Proinflammatory proliferation and differentiation, pyrogenic	[3,4]
**IL-2**	$CD4^+$ and $CD8^+$ activated T cells, dendritic cells, NK cells	$CD4^+$ and $CD8^+$ T cells, B and NK cells	Proliferation of effector T and B cells, development of Treg cells, B and NK cells stimulation, proliferation and cytokine production in innate lymphoid cells	[5,6]
**IL-6**	T and B cells, EC, monocytes, macrophages, granulocytes	Leukocytes, T and B cells, haematopoietic cells	Leukocyte trafficking and activation; T and B cell differentiation, activation and survival;	[7]
**IL-8**	Monocytes, macrophages, neutrophils, lymphocytes, EC, fibroblasts	T cells, neutrophils, NK cells, monocytes, endothelial cells, eosinphils, mast cells	Chemoattractant for T cells, neutrophils, NK cells, basophils, eosinophils, mobilization of haemotopoietic stem cells, angiogenesis	[8]
**IL-10**	T and B cells, monocytes, macrophages and dendritic cells	Macrophages, monocytes, T and B cells, NK cells, DC	Immunosuppressive effect through antigen presenting cells,	[9]
**TNFα**	Activated macrophages, monocytes, CD^+ T cells, B cells, NK cells, neutrophils	Nucleated cells	Host defense, dual role as proinflammatory and immunosuppressive mediator	[10]
**IFNγ**	Macrophages, cytotoxic T lymphocytes, B cells, NK cells	Epithelial cells, macrophages, dendritic cells, NK cells, T and B cells	Antiviral properties, promotion of cytotoxic activity, T_H1 differentiation, upregulation of MHC class I and II, inhibition of cell proliferation, proapoptotic effects	[11]
**GRO (growth related oncogene) CXCL1**	Macrophages, neutrophils, epithelial cells	Leukocytes	Chemotaxis, inflammation, angiogenesis, tumor cell transformation, wound healing	[12,13]
**IP-10 (IFNγ-inducible protein) CXCL10**	T and B cells, monocytes, EC, neutrophils, epithelium	Activated T and B cells, NK cells, dendritic cells, macrophages	Chemotaxis, proinflammatory, modulate angiogenesis and wound healing	[14-16]

**FGF2**	Cells of inflammation and immunity, T cells, mononuclear phagocytes, and EC	Autocrine action, EC, variety of tissues	Inflammation, neovascularisation, angiogenesis, wound healing	[17-20]

Supplementary Table S4: Overview of cytokine produced, their cell source, targets, and functions.

References:

1. Akdis, M.; Aab, A.; Altunbulakli, C.; Azkur, K.; Costa, R.A.; Crameri, R.; Duan, S.; Eiwegger, T.; Eljaszewicz, A.; Ferstl, R., et al. Interleukins (from IL-1 to IL-38), interferons, transforming growth factor beta, and TNF-alpha: Receptors, functions, and roles in diseases. *The Journal of allergy and clinical immunology* **2016**, *138*, 984-1010, doi:10.1016/j.jaci.2016.06.033.
2. Basu, S.; Hodgson, G.; Katz, M.; Dunn, A.R. Evaluation of role of G-CSF in the production, survival, and release of neutrophils from bone marrow into circulation. *Blood* **2002**, *100*, 854-861, doi:10.1182/blood.v100.3.854.
3. Dinarello, C.A.; Renfer, L.; Wolff, S.M. Human leukocytic pyrogen: purification and development of a radioimmunoassay. *Proc Natl Acad Sci U S A* **1977**, *74*, 4624-4627, doi:10.1073/pnas.74.10.4624.
4. Garlanda, C.; Dinarello, C.A.; Mantovani, A. The interleukin-1 family: back to the future. *Immunity* **2013**, *39*, 1003-1018, doi:10.1016/j.immuni.2013.11.010.
5. Morgan, D.A.; Ruscetti, F.W.; Gallo, R. Selective in vitro growth of T lymphocytes from normal human bone marrows. *Science* **1976**, *193*, 1007-1008, doi:10.1126/science.181845.
6. Roediger, B.; Kyle, R.; Tay, S.S.; Mitchell, A.J.; Bolton, H.A.; Guy, T.V.; Tan, S.Y.; Forbes-Blom, E.; Tong, P.L.; Koller, Y., et al. IL-2 is a critical regulator of group 2 innate lymphoid cell function during pulmonary inflammation. *The Journal of allergy and clinical immunology* **2015**, *136*, 1653-1663 e1657, doi:10.1016/j.jaci.2015.03.043.
7. Uyttenhove, C.; Coulie, P.G.; Van Snick, J. T cell growth and differentiation induced by interleukin-HP1/IL-6, the murine hybridoma/plasmacytoma growth factor. *J Exp Med* **1988**, *167*, 1417-1427, doi:10.1084/jem.167.4.1417.
8. Coelho, A.L.; Hogaboam, C.M.; Kunkel, S.L. Chemokines provide the sustained inflammatory bridge between innate and acquired immunity. *Cytokine Growth Factor Rev* **2005**, *16*, 553-560, doi:10.1016/j.cytogfr.2005.03.004.
9. Stanic, B.; van de Veen, W.; Wirz, O.F.; Ruckert, B.; Morita, H.; Sollner, S.; Akdis, C.A.; Akdis, M. IL-10-overexpressing B cells regulate innate and adaptive immune responses. *The Journal of allergy and clinical immunology* **2015**, *135*, 771-780 e778, doi:10.1016/j.jaci.2014.07.041.
10. Brown, S.D.; Brown, L.A.; Stephenson, S.; Dodds, J.C.; Douglas, S.L.; Qu, H.; Fitzpatrick, A.M. Characterization of a high TNF-alpha phenotype in children with moderate-to-severe asthma. *The Journal of allergy and clinical immunology* **2015**, *135*, 1651-1654, doi:10.1016/j.jaci.2014.08.054.
11. Akkoc, T.; de Koning, P.J.; Ruckert, B.; Barlan, I.; Akdis, M.; Akdis, C.A. Increased activation-induced cell death of high IFN-gamma-producing T(H)1 cells as a mechanism of T(H)2 predominance in atopic diseases. *The Journal of allergy and clinical immunology* **2008**, *121*, 652-658 e651, doi:10.1016/j.jaci.2007.12.1171.
12. Bhat, K.; Sarkissyan, M.; Wu, Y.; Vadgama, J.V. GROalpha overexpression drives cell migration and invasion in triple negative breast cancer cells. *Oncology reports* **2017**, *38*, 21-30, doi:10.3892/or.2017.5668.
13. Fujiwara, K.; Matsukawa, A.; Ohkawara, S.; Takagi, K.; Yoshinaga, M. Functional distinction between CXC chemokines, interleukin-8 (IL-8), and growth related oncogene (GRO)alpha in neutrophil infiltration. *Lab Invest* **2002**, *82*, 15-23.
14. Neville, L.F.; Mathiak, G.; Bagasra, O. The immunobiology of interferon-gamma inducible protein 10 kD (IP-10): a novel, pleiotropic member of the C-X-C chemokine superfamily. *Cytokine Growth Factor Rev* **1997**, *8*, 207-219.
15. Liu, M.; Guo, S.; Hibbert, J.M.; Jain, V.; Singh, N.; Wilson, N.O.; Stiles, J.K. CXCL10/IP-10 in infectious diseases pathogenesis and potential therapeutic implications. *Cytokine Growth Factor Rev* **2011**, *22*, 121-130, doi:10.1016/j.cytogfr.2011.06.001.

16. Liu, M.; Guo, S.; Stiles, J.K. The emerging role of CXCL10 in cancer (Review). *Oncol Lett* **2011**, *2*, 583-589, doi:10.3892/ol.2011.300.
17. Rusnati, M.; Camozzi, M.; Moroni, E.; Bottazzi, B.; Peri, G.; Indraccolo, S.; Amadori, A.; Mantovani, A.; Presta, M. Selective recognition of fibroblast growth factor-2 by the long pentraxin PTX3 inhibits angiogenesis. *Blood* **2004**, *104*, 92-99, doi:10.1182/blood-2003-10-3433.
18. Delrieu, I. The high molecular weight isoforms of basic fibroblast growth factor (FGF-2): an insight into an intracrine mechanism. *FEBS letters* **2000**, *468*, 6-10, doi:10.1016/s0014-5793(00)01189-3.
19. Yun, Y.R.; Won, J.E.; Jeon, E.; Lee, S.; Kang, W.; Jo, H.; Jang, J.H.; Shin, U.S.; Kim, H.W. Fibroblast growth factors: biology, function, and application for tissue regeneration. *J Tissue Eng* **2010**, *2010*, 218142, doi:10.4061/2010/218142.
20. Seghezzi, G.; Patel, S.; Ren, C.J.; Gualandris, A.; Pintucci, G.; Robbins, E.S.; Shapiro, R.L.; Galloway, A.C.; Rifkin, D.B.; Mignatti, P. Fibroblast growth factor-2 (FGF-2) induces vascular endothelial growth factor (VEGF) expression in the endothelial cells of forming capillaries: an autocrine mechanism contributing to angiogenesis. *J Cell Biol* **1998**, *141*, 1659-1673, doi:10.1083/jcb.141.7.1659.
